# The Italian record of the Cretaceous shark, *Ptychodus latissimus* Agassiz, 1835 (Chondrichthyes; Elasmobranchii)

**DOI:** 10.7717/peerj.10167

**Published:** 2020-11-24

**Authors:** Manuel Amadori, Jacopo Amalfitano, Luca Giusberti, Eliana Fornaciari, Giorgio Carnevale, Juergen Kriwet

**Affiliations:** 1 University of Vienna, Department of Paleontology, Vienna, Austria; 2 Dipartimento di Geoscienze, Università degli Studi di Padova, Padova, Italy; 3 Dipartimento di Scienze della Terra, Università degli Studi di Torino, Torino, Italy

**Keywords:** Fossil sharks, Taxonomy, Heterodonty, Durophagy, Upper Cretaceous, Veneto region

## Abstract

Associated and isolated teeth of the extinct elasmobranch *Ptychodus latissimus* Agassiz, 1835 from the Upper Cretaceous Scaglia Rossa pelagic limestone of northern Italy are described and discussed here in detail for the first time. The dentition of this widely distributed species consists of low-crowned molariform teeth that exhibit marked and strong occlusal ornamentations suitable for crushing hard-shelled prey. The associated tooth sets and isolated teeth analyzed here are heterogeneous in size and crown outline, but unambiguously belong to a single species. Re-examination of this Italian material consisting of ca. 30 specimens mostly coming from historical collections allows for a rigorous assessment of the intraspecific variability of *P. latissimus*, including the identification of three different tooth “morphotypes” based on their positions within the jaws. The relatively flat crowns and occlusal sharp and thick ridges indicate a high adaptation for crushing hard-shelled prey in *P. latissimus* indicating that the durophagous adaptations of this species were certainly more pronounced than in all other species of *Ptychodus*. We hypothesize that *P. latissimus* was a third-level predator occupying habitats with abundant thick-shelled prey, such as inoceramid bivalves and ammonites.

## Introduction

*Ptychodus* Agassiz, 1834 is a genus of Cretaceous elasmobranchs ranging from the Albian to the Campanian with worldwide distribution (Woodward, 1912; Cappetta, 2012). The fossil record of this enigmatic predator mainly includes isolated teeth, very rare associated, but also articulated dentitions and mineralized cartilaginous elements (Nicolis, 1889; Woodward, 1889; Tan, 1949; MacLeod, 1982; Everhart & Caggiano, 2004; Shimada et al., 2010; Cappetta, 2012; Shimada, 2012; Diedrich, 2013; Hamm, 2017; Amadori et al., 2019a, 2019b, 2020; Jambura & Kriwet, 2020). *Ptychodus* possessed polygonal, molariform teeth arranged in dental plates suitable for crushing shelled preys (Williston, 1900a, 1900b; Woodward, 1912; Cappetta, 2012; Shimada, 2012; Diedrich, 2013; Amadori et al., 2019b, 2020). Marked interspecific and intraspecific variability characterizes the dental morphologies of this durophagous elasmobranch of unknown affinities, making its already problematic dental-based taxonomy and systematic relationships even more difficult (Nicholls, 2010; Shimada, 2012; Amadori et al., 2019b, 2020). Different degrees of heterodonty have been also recently recognized based on comparisons of articulated tooth sets (see Woodward, 1912; Shimada, 2012; Amadori et al., 2020). In general, *Ptychodus* exhibits relatively flat or markedly cusped dental crowns, which could suggest opportunistic or more specialized feeding adaptations, respectively (Shimada, 2012; Diedrich, 2013; Amadori et al., 2019b, 2020).

*Ptychodus latissimus* Agassiz, 1835 is a low-crowned species characterized by flat or gently raised dental crown exhibiting very thick occlusal ornamentation (see Leriche, 1906; Woodward, 1912; Herman, 1977; Diedrich, 2013; Amadori et al., 2020). Numerous isolated teeth and rare associated finds of this species are well known from the upper Turonian to lower Coniacian of Europe (see Leriche, 1906; Dibley, 1911; Woodward, 1889, 1912; Vullo & Arnaud, 2009; Diedrich, 2013). *Ptychodus latissimus* was rather rare in the West Interior Sea with a few dozens of isolated teeth reported from the lower Coniacian of Texas (see Hamm, 2020). Rare isolated finds of *P. latissimus* also were reported from the upper Turonian of Angola and lower Coniacian of Japan (Antunes & Cappetta, 2002; Tan, 1949). Various systematic, taxonomic and nomenclatural issues related to this low-crowned taxon have been recently disentangled, also establishing the main diagnostic dental features useful to unambiguously distinguish *P. latissimus* from the morphologically similar species *P. mediterraneus* and *P. polygyrus* (Amadori et al., 2020). Associated tooth sets and isolated teeth of *Ptychodus latissimus* from Italy are carefully documented and discussed in this study to evaluate the intraspecific variability of this low-crowned taxon in detail and further support the formal revision of the species proposed by Amadori et al. (2020).

In addition, a preliminary discussion on the feeding specialization of *P. latissimus* based on its typical tooth ornamentation patterns is provided herein.

## Historical Background

**Figure 1:**
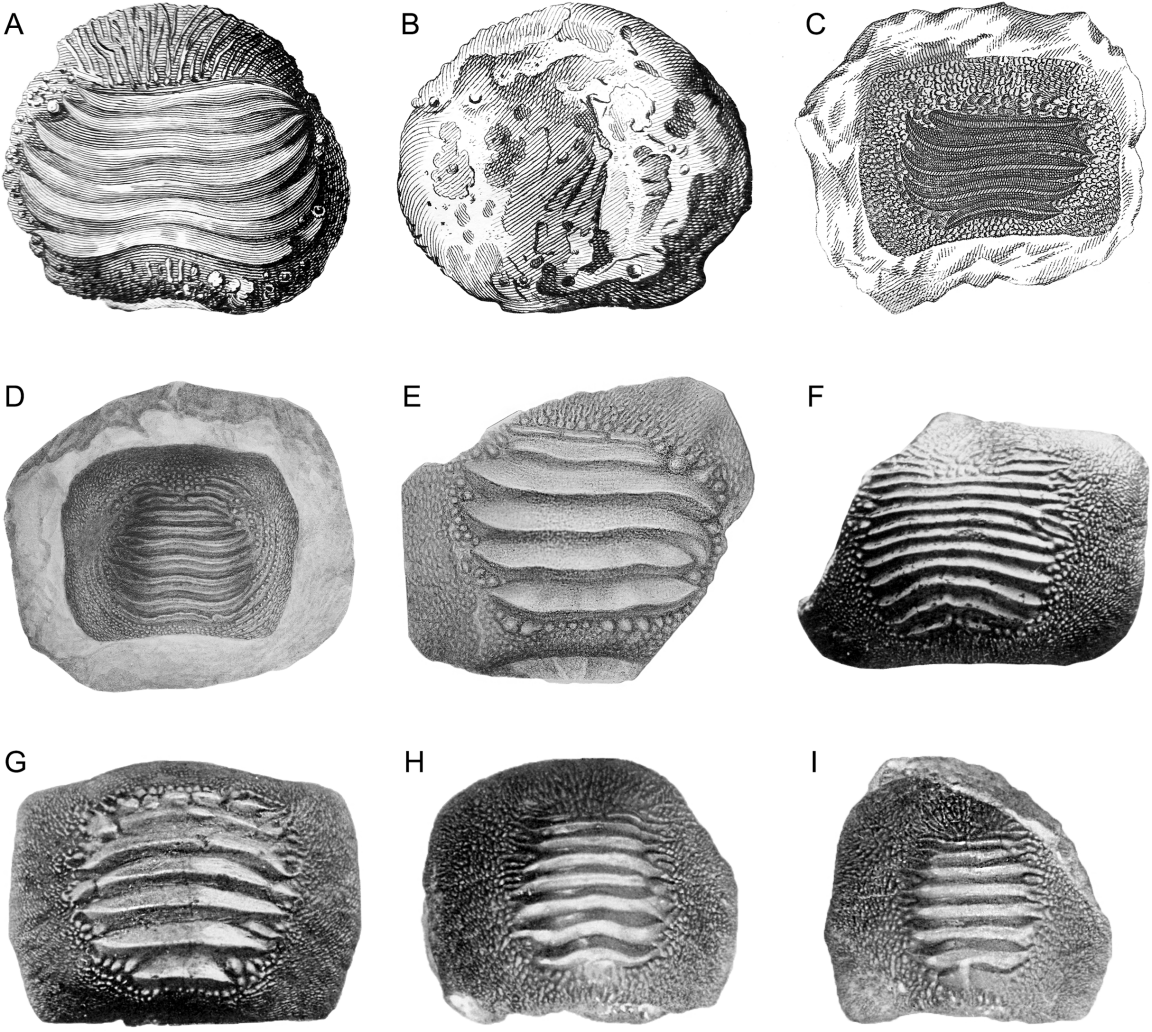
Historical finds of *Ptychodus* from Italy assigned herein to *P. latissimus* Agassiz 1835 in occlusal (A, C–M), lateral (N) and inferior (B) views. (A and B) First Italian illustration of a tooth of *Ptychodus* (after “Magazzino universale aperto per l’utilità, e il diletto di tutti”, 1751). (C) Isolated tooth coming from the surroundings of Castellavazzo (Belluno; after Catullo, 1820). (D and E) Isolated teeth originally figured in a draft of an unpublished plate by Achille De Zigno (see Amadori et al., 2019a). The specimen (D) (MGP-PD 27249) comes from Castellavazzo (Belluno), whereas the specimen (E) (MGP-PD 6741) comes from Prun (Verona). (F–I) Specimens illustrated in the “Catalogo dei pesci fossili delle Tre Venezie” by D’Erasmo (1922). The specimen (F) (MGP-PD 12202) comes from Valdagno (Vicenza), the tooth (G) is from Mel (Belluno), whereas (H) and (I) are from Novale (Vicenza). Specimens (G–I) are presently lost.

**Figure 2:**
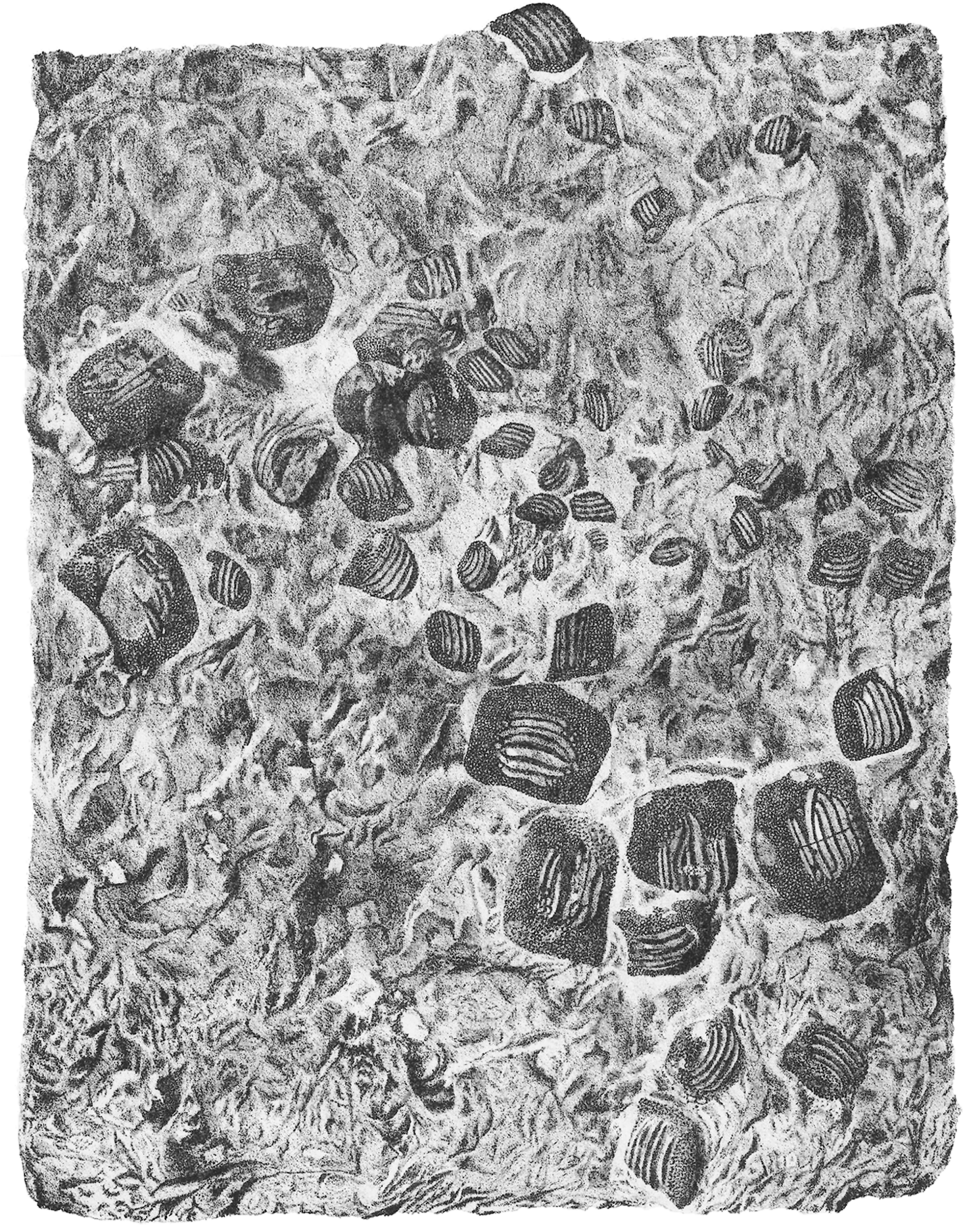
The original plate of Nicolis (1889) illustrating the slab MCSNV v.1612 with the associated tooth set of *Ptychodus latissimus* Agassiz, 1835 coming from Prun (Verona province). Courtesy of Accademia di Agricoltura Scienze e Lettere di Verona.

Among the first remains of *Ptychodus latissimus* recovered in Italy are both isolated and associated teeth reported from the Upper Cretaceous of the Belluno province (Brocchi, 1814; Catullo, 1818, 1820; see also Amadori et al., 2019a). The first illustration of an isolated tooth of *Ptychodus* in Italy, however, already was presented in 1751 in the oeuvre “Magazzino universale aperto per l’utilità, e il diletto di tutti” published in Venice and was identified as ‘bizzarre fossil’. The tooth, whose provenance is unknown, shows typical features of *P. latissimus* (see Figs. 1A and 1B) and was also cited by Brocchi (1814) as ‘fish fossil palate’. Later, Catullo (1820, 1827) figured a polygonal tooth of “*Diodon*” (Fig. 1C) from the surroundings of Castellavazzo (Belluno province), which he subsequently ascribed to *P. latissimus* (see Catullo, 1842: 10). Bassani (1886) documented a broken tooth of this low-crowned species, together with other isolated fish teeth, from the same locality. The associated tooth set from Prun (Verona province) described herein (see below) was originally reported by Pellegrini (1883: 145*)* and later figured by Nicolis (1889; Fig. 2). This tooth set was subsequently mentioned and figured (Canavari, 1916; Coggi, 1964; Aspes & Zorzin, 1984; Zorzin, 2001, 2017; Zorzin & Vaccari, 2005), although it was never examined in detail. Remains of *P. latissimus* figured by Bassani (1886) and Nicolis (1889) were included, together with other isolated teeth (e.g., Figs. 1F–1I) at least partially attributable to *P. latissimus*, in the last comprehensive revision of the fossil fish record from northeastern Italy by D’Erasmo (1922). Occurrences of *P. latissimus* and other species of the genus outside northeastern Italy are sparse and/or poorly documented (see also Amadori et al., 2019a, 2019b, 2020). Sacco (1905) reported several specimens of *Ptychodus* from the surroundings of Vernasca (Piacenza province, Italy). In particular, the author figured three small fragmentary finds (see Sacco, 1905: pl. 8, figs. 11a–c), assigned to *P. latissimus* despite just consisting of tooth crown fragments of three to six thick and marked ridges exhibited in occlusal view. Their marginal ornamentation is hardly recognizable due to the poor preservation, or the complete absence, of the tooth margins. Only one of these fragments exhibits marked abrasions on the posterior ridges. Sacco (1905: 255*)* hypothesized a Cenomanian-“Danian” age for the “argille scagliose” (“scaly clays”) from which all the finds come, also specifying that the teeth of *Ptychodus*, together with those of various other fossil fishes, are prevalent in the Campanian. A fragmentary tooth from the eastern Madonie Mountains (Palermo province, central-northern Sicily) was assigned to *P. latissimus* by Coggi (1964: 125*)* despite faint resemblances to *P. mediterraneus*. The examination of its dental features is enough to confirm the original attribution by Coggi (1964: text fig. 1; see also Amadori et al., 2020). In occlusal view, the tooth described by Coggi (1964) exhibits five thick ridges clearly separated from an incomplete marginal area, originally hidden by limestone matrix. In lateral view, the outline of the crown is medially convex. The micropaleontological content of the matrix in which this find is embedded mainly consists of planktic foraminifera indicating a Turonian age (Coggi, 1964). Another isolated tooth from Upper Cretaceous brown limestones of Contrada S. Nicola (Messina province, Italy), originally identified as *P. latissimus*, recently has been reassigned to *P. mediterraneus* (see Seguenza, 1900; Amadori et al., 2020).

## Geological Setting

**Figure 3:**
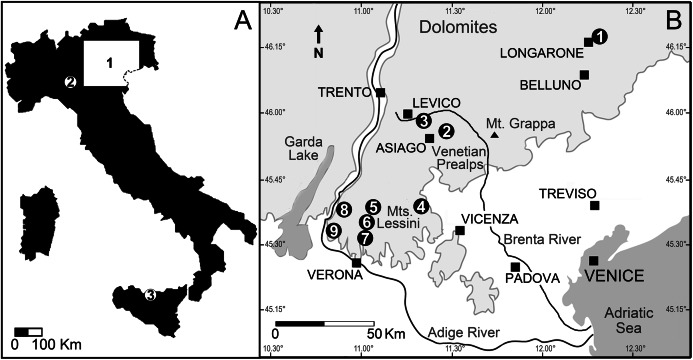
Location of the Italian sites that yielded remains of the Cretaceous shark *Ptychodus latissimus*. (A) Sketch map of Italy: (1) northeastern Italy; (2) surroundings of Vernasca, Piacenza; (3) Madonie Mountains, Sicily. (B) Blow-up of northeastern Italy area with indication of the Scaglia Rossa sites that yielded the fossils studied herein (modified from Amadori et al., 2020): (1) Castellavazzo (Belluno); (2) Gallio (Vicenza); (3) surroundings of Levico (Trento); (4) Valdagno (Vicenza); (5) surroundings of S. Anna d’Alfaedo (Verona); (6) Prun (Verona); (7) Negrar (Verona); (8) Breonio (Verona); (9) Mazzurega (Verona). Light gray, mountain landscapes.

Most of the Italian specimens of *Ptychodus latissimus* Agassiz, 1835 described herein come from the “Lastame” of the Lessini Mountains (Verona province, Italy) and the “Pietra di Castellavazzo” exposed in the Castellavazzo area (Belluno province, Italy). These two peculiar nodular/subnodular stratigraphic intervals pertain to the Upper Cretaceous pelagic to hemipelagic succession of the Scaglia Rossa Formation of northeastern Italy (see also Bassani, 1886, 1888; Trevisani & Cestari, 2007; Roghi & Romano, 2009; Amalfitano et al., 2017a, 2017c; Amadori et al., 2019b, 2020; see Fig. 3). Both lithozones have a quite heterogeneous paleontological content of marine vertebrates among which chondrichthyans represent the largest part of the fossil assemblages (Cigala-Fulgosi et al., 1980; Dalla Vecchia et al., 2005; Trevisani & Cestari, 2007; Roghi, 2010; Palci, Caldwell & Papazzoni, 2013; Amalfitano et al., 2017a, 2017b, 2017c, 2019a; Amadori et al., 2019a, 2019b, 2020; Larocca Conte et al., 2019). A stratigraphic revision of the “Lastame” lithozone, which is assumed to span from early Turonian to early Santonian is currently in progress (Amalfitano et al., 2019a). However, the calcareous nannofossil assemblages of the matrix of specimens of *Ptychodus* and other sharks coming from “Lastame” that have been examined up to now, are indicative of the UC7-UC9 zones of Burnett (1999) (see Amalfitano et al., 2019a, 2019b; Amadori et al., 2019b, 2020), suggesting an early-late Turonian age (Ogg & Hinnov, 2012) for the fossiliferous beds of “Lastame”. Although the stratigraphic range of “Pietra di Castellavazzo” is still debated, historical works advocate a late Turonian-late Campanian age for the exposures in the Castellavazzo area (see Larocca Conte et al., 2019). The only fossil that has been confidently dated from Castellavazzo beds so far is a lamniform shark, whose matrix yielded a planktic foraminiferal assemblage indicating an early Santonian age (see Larocca Conte et al., 2019).

## Materials and Methods

### Materials

For the purposes of the present work, we recovered all available specimens coming from the deposits of the Scaglia Rossa Formation of Veneto and Trentino-Alto Adige regions in northeastern Italy. Most of them belong to historical collections housed in Italian and Austrian museums. Some specimens were previously undescribed or recently discovered in unsorted or only partially known collections (Amadori et al., 2019a). The rare and poorly preserved specimens of *P. latissimus* coming from northwestern Italy and central-southern Italy were not included in the present study. The majority of the specimens investigated are housed in the Museo Civico di Storia Naturale di Verona (catalogue numbers: MCSNV v.1612, MCSNV v.12510, MCSNV v.12511, MCSNV v.12513, MCSNV v.12515, MCSNV v.12516 and MCSNV v.12517) and Museo di Geologia e Paleontologia dell’Università di Padova (catalogue numbers: MGP-PD 3803, MGP-PD 3804, MGP-PD 6729, MGP-PD 6741, MGP-PD 6742, MGP-PD 7347, MGP-PD 8491, MGP-PD 8495, MGP-PD 12201, MGP-PD 12202, MGP-PD 12203, MGP-PD 14028, MGP-PD 14030, MGP-PD 23538, MGP-PD 23540 and MGP-PD 27249). Other fossils are deposited in the Museo Civico di Rovereto, Trento (catalogue numbers: MCR FO 00662 and MCR FO 00663E; Amadori et al., 2019a), Museo di Storia Naturale dell’Università di Pisa (catalogue number: MSNUP 272), Museo Geopaleontologico di Camposilvano, Verona (catalogue number: MGC VR 47890) and Museo Civico D. Dal Lago, Valdagno (catalogue number: MCV 779), Vicenza, Italy. Some Italian specimens (NHMW 8543α-γ) also are housed in the Naturhistorisches Museum, Vienna, Austria.

### Methods

High quality photos of the specimens were obtained using a Nikon D810 camera with mounted 60–90 mm lens and a Canon PowerShot SX720 HS. The species-specific features on the dental crown of some specimens were enhanced using the “smoking” technique (Scovil, 1996). The illustrative drawings and images of the finds were prepared using GIMP (v. 2.8.16), Photoshop CS5 (v.12.0 x32) and Inkscape (v. 0.92) software packages, while the teeth were measured with the image analysis package Image J (v. 1.6; Schneider, Rasband & Eliceiri, 2012). Measurements were approximated avoiding decimals.

Statistical analyses were performed using the software package Past 3.26 (Hammer, Harper & Ryan, 2001) to preliminarily investigate the morphological variability inside the tooth set MCSNV v. 1612. The PCA protocol by Marramà & Kriwet (2017) was applied to the analyses. The logtransformation is the only data treatment used here to overcome the problem of the non-normal distribution of data by un-stretching large scales of values (see Marramà & Kriwet, 2017 and references therein), by analyzing teeth coming from a single individual. For PCA parameters see the Supplemental Data.

Smear slides were prepared through matrix powder obtained from the specimens for analyses of calcareous nannofossils (see Amalfitano et al., 2017c; Amadori et al., 2019b, 2020).

The anatomical and odontological terminology mostly follows Cappetta (2012), Shimada (2012), Hamm (2017, 2019) and Amadori et al. (2020). The synonymy lists follow the standards proposed by Matthews (1973), Bengtson (1988) and Sigovini, Keppel & Tagliapietra (2016).

## Results

**Systematic paleontology**

Class CHONDRICHTHYES Huxley, 1880

Subclass ELASMOBRANCHII Bonaparte, 1838

Order PTYCHODONTIFORMES Hamm, 2019

Family †PTYCHODONTIDAE Jaekel, 1898

Genus †*PTYCHODUS* Agassiz, 1834

***Type species***. *Ptychodus schlotheimii* Agassiz, 1834 (nomen oblitum), senior synonym of *Ptychodus latissimus* Agassiz, 1835 (nomen protectum). See Giusberti et al. (2018).

***Diagnosis***. See Woodward (1912) and Jambura & Kriwet (2020).

†*Ptychodus latissimus* Agassiz, 1835

(Figs. 1, 2 and 4–14)

**Figure 4:**
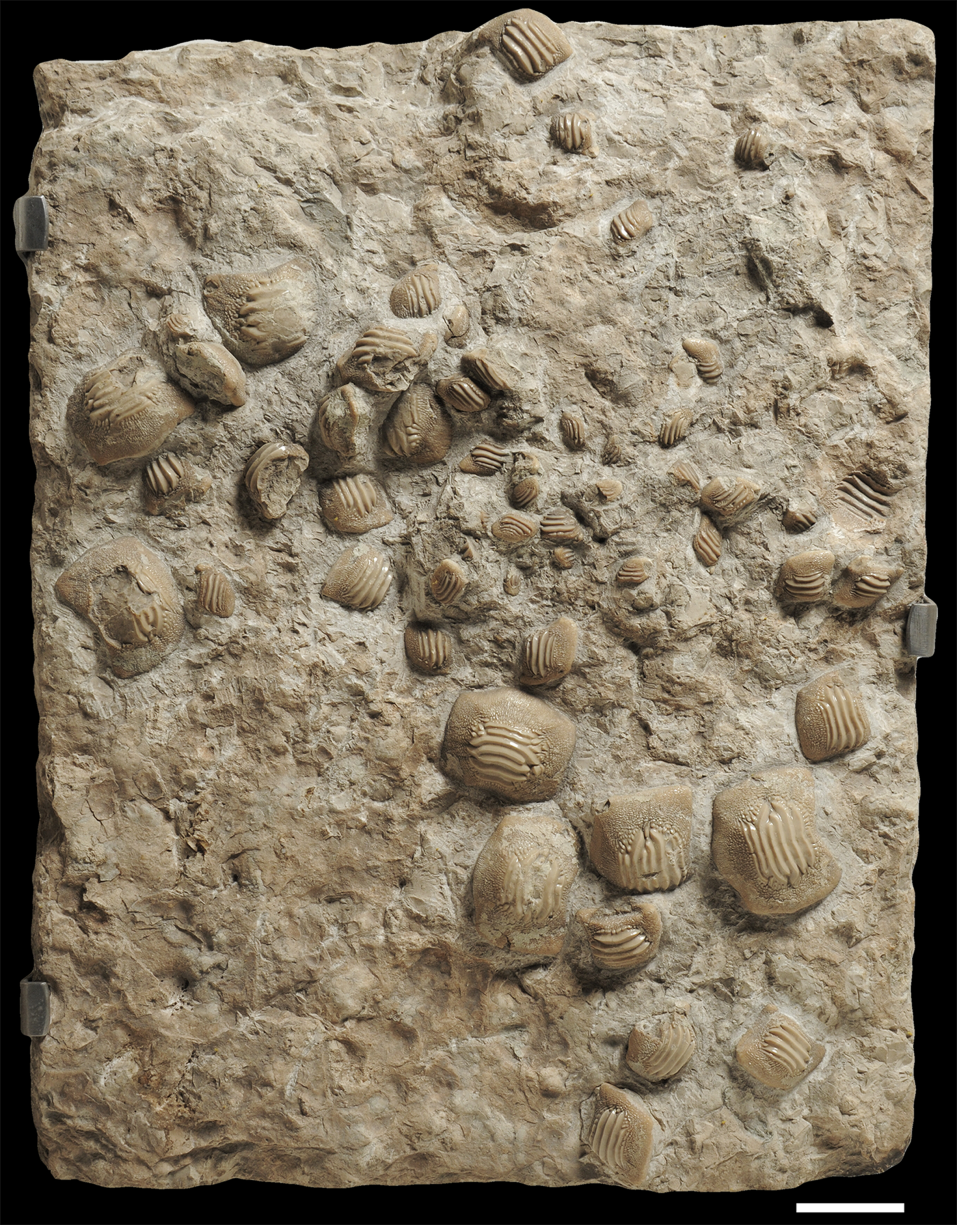
The associated tooth set MCSNV v.1612 from Prun (Verona province) that includes about 52 teeth of *Ptychodus latissimus* Agassiz, 1835. Scale bar equals 50 mm.

**Figure 5:**
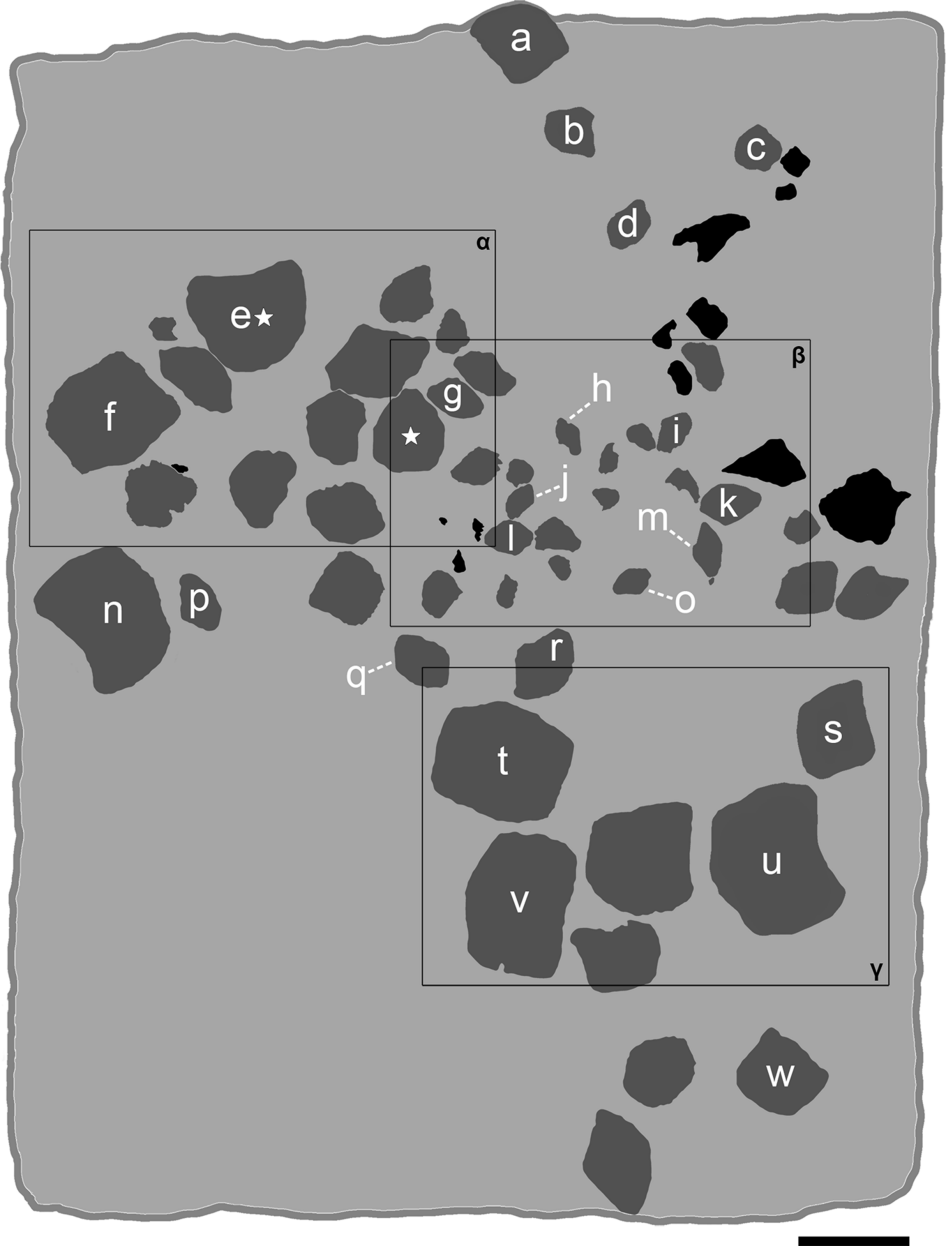
Interpretative line drawing of the associated specimen MCSNV v.1612 (light gray, matrix; dark gray, teeth preserved; black areas, tooth imprints; white stars, worn teeth). The black rectangles show the areas illustrated in the detail figures (α, see Fig. 6; β, see Fig. 7; γ, see Fig. 8). (a) MCSNV v.1612a; (b) MCSNV v.1612b; (c) MCSNV v.1612c; (d) MCSNV v.1612d; (e) MCSNV v.1612e; (f) MCSNV v.1612f; (g) MCSNV v.1612g; (h) MCSNV v.1612h; (i) MCSNV v.1612i; (j) MCSNV v.1612j; (k) MCSNV v.1612k; (l) MCSNV v.1612l; (m) MCSNV v.1612m; (n) MCSNV v.1612n; (o) MCSNV v.1612o; (p) MCSNV v.1612p; (q) MCSNV v.1612q; (r) MCSNV v.1612r; (s) MCSNV v.1612s; (t) MCSNV v.1612t; (u) MCSNV v.1612u; (v) MCSNV v.1612v; (w) MCSNV v.1612w. Scale bar equals 50 mm.

**Figure 6:**
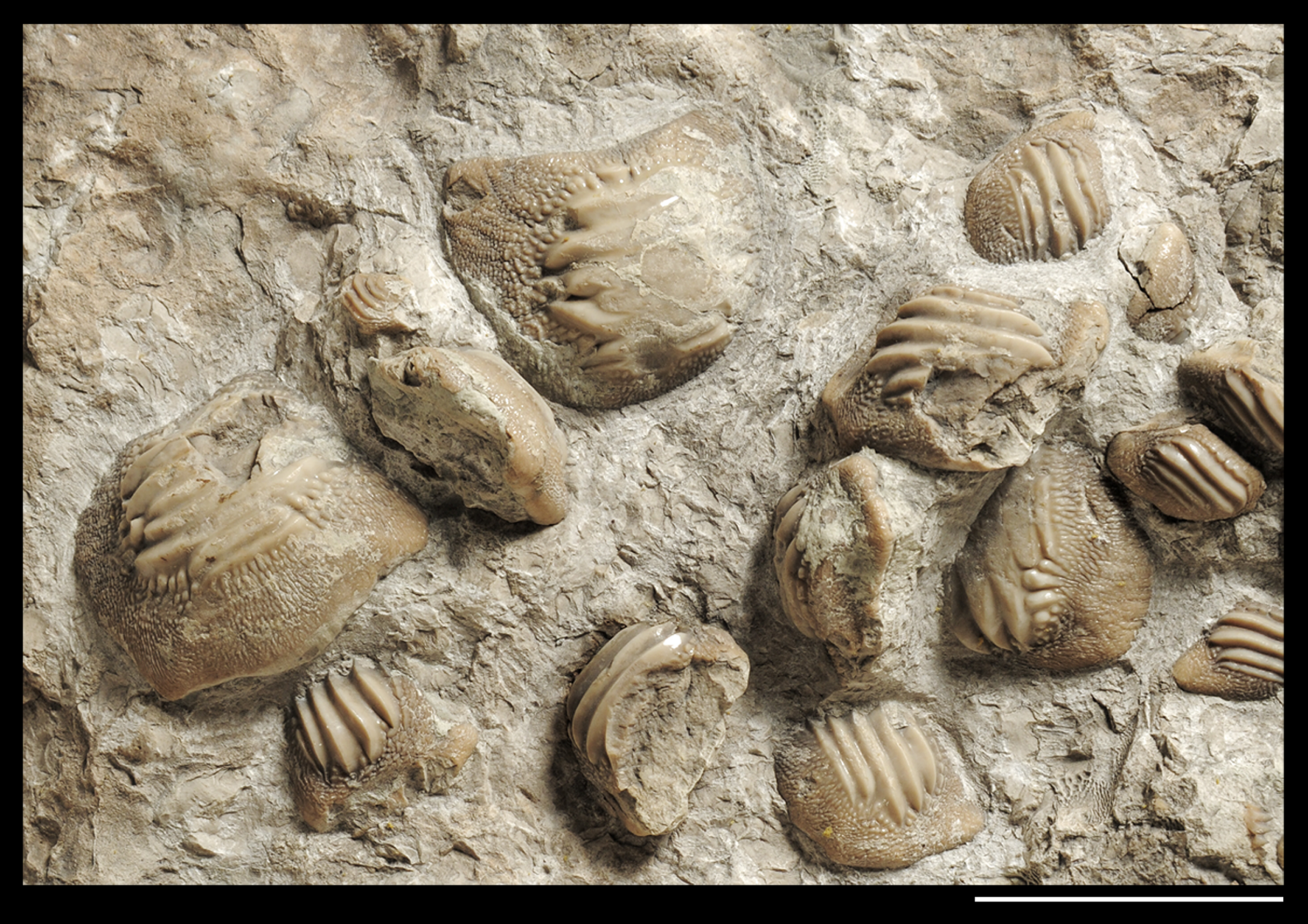
Detail of the slab MCSNV v.1612 exhibiting large worn teeth of *Ptychodus latissimus* Agassiz, 1835 (see also α in Fig. 5). Scale bar equals 50 mm.

**Figure 7:**
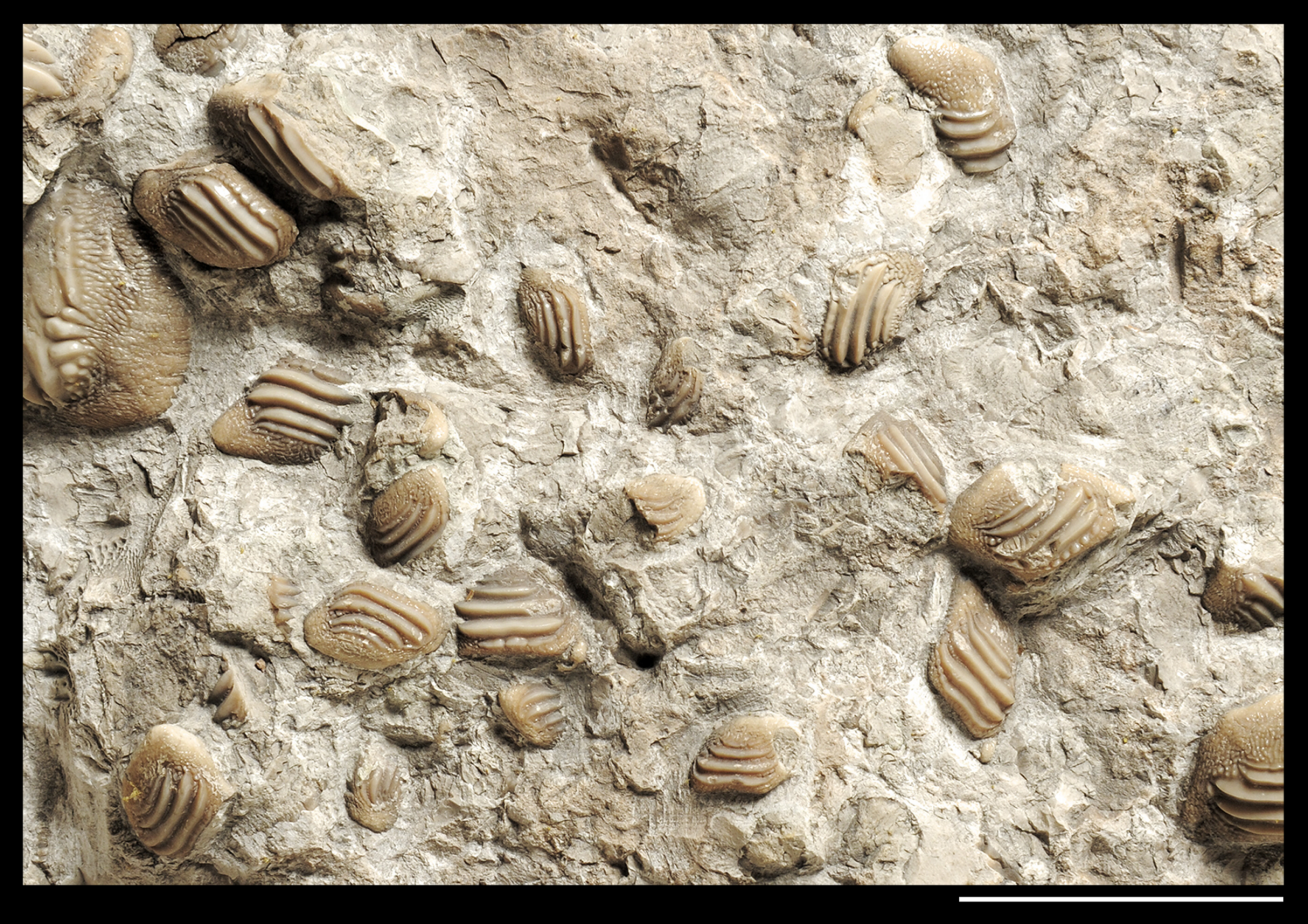
Detail of the slab MCSNV v.1612 exhibiting teeth of *Ptychodus latissimus* Agassiz, 1835 characterized by various sizes and asymmetric morphologies (see also β in Fig. 5). Scale bar equals 50 mm.

**Figure 8:**
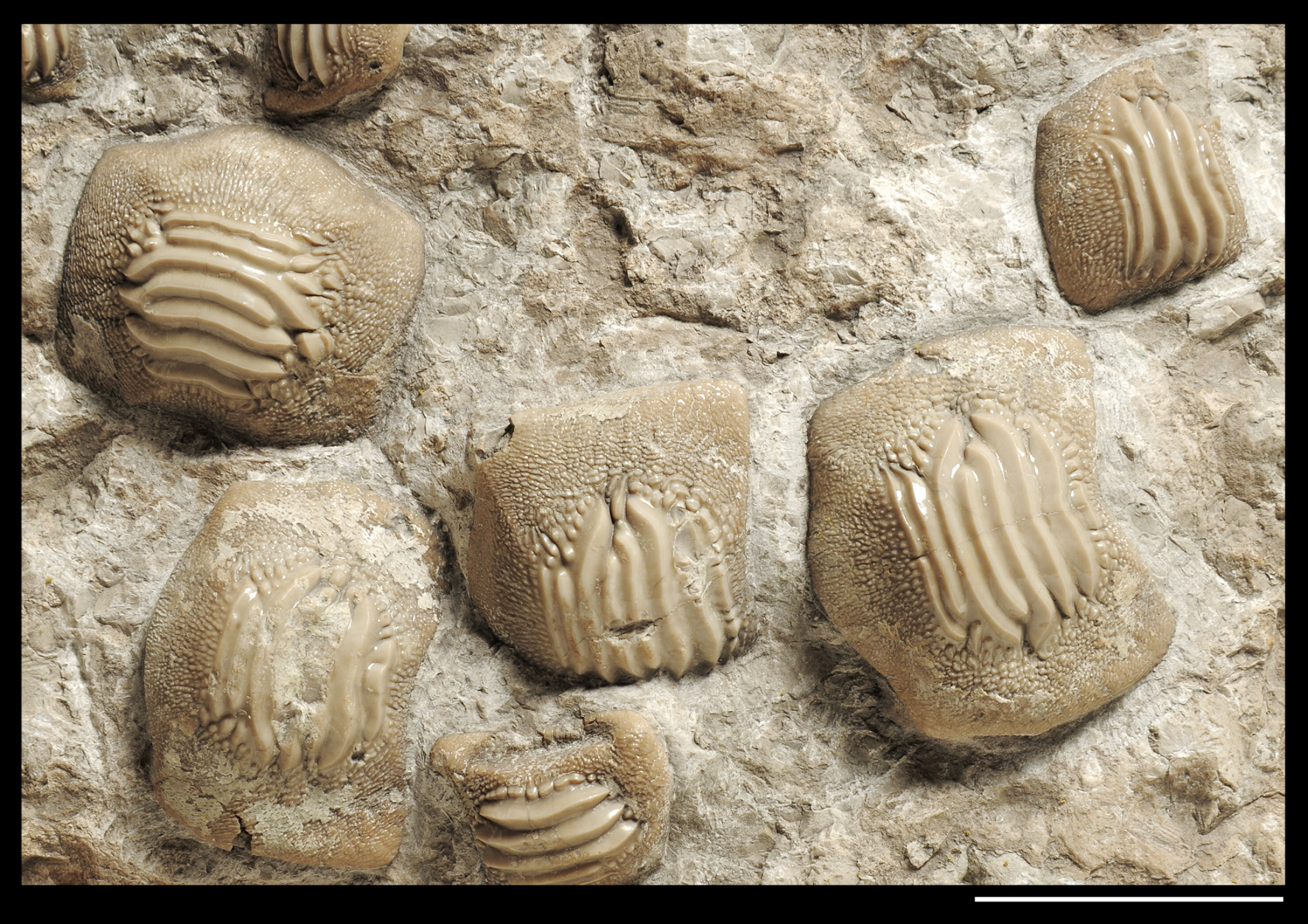
Detail of the slab MCSNV v.1612 mostly exhibiting large teeth of *Ptychodus latissimus* Agassiz, 1835 characterized by symmetric dental crowns (see also γ in Fig. 5). Scale bar equals 50 mm.

**Figure 9:**
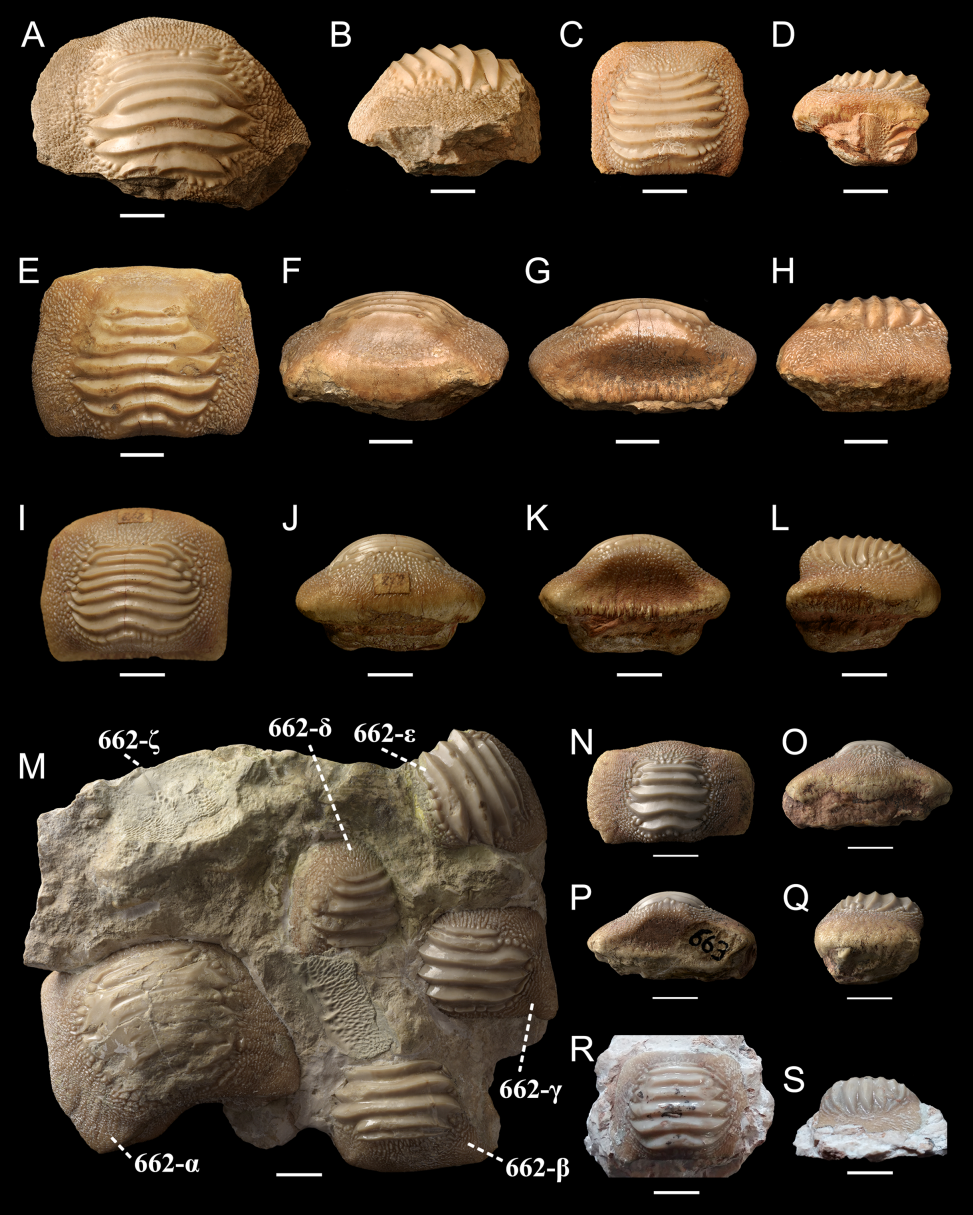
Isolated (A-L, N-S) and associated (M) specimens of *Ptychodus latissimus* Agassiz, 1835 from the northeastern Italy in occlusal (A, C, E, I, M, N and R), anterior (F, J and O), posterior (G, K and P) and lateral (B, D, H, L, Q and S) views. (A and B) Symphyseal tooth MCSNV v.12510. (C and D) Lateral tooth MCSNV v.12513α. (E–H) Symphyseal tooth MCSNV v.12516α. (I–L) Symphyseal tooth MSNUP 272. (M) Associated tooth set MCR FO 00662 preserving six teeth (662-α, 662-β, 662-γ, 662-δ, 662-ε, 662-ζ). (N–Q) Symphyseal tooth MCR FO 00663E. (R and S) Lateral tooth MGC VR 47890. Scale bars equal 10 mm.

**Figure 10:**
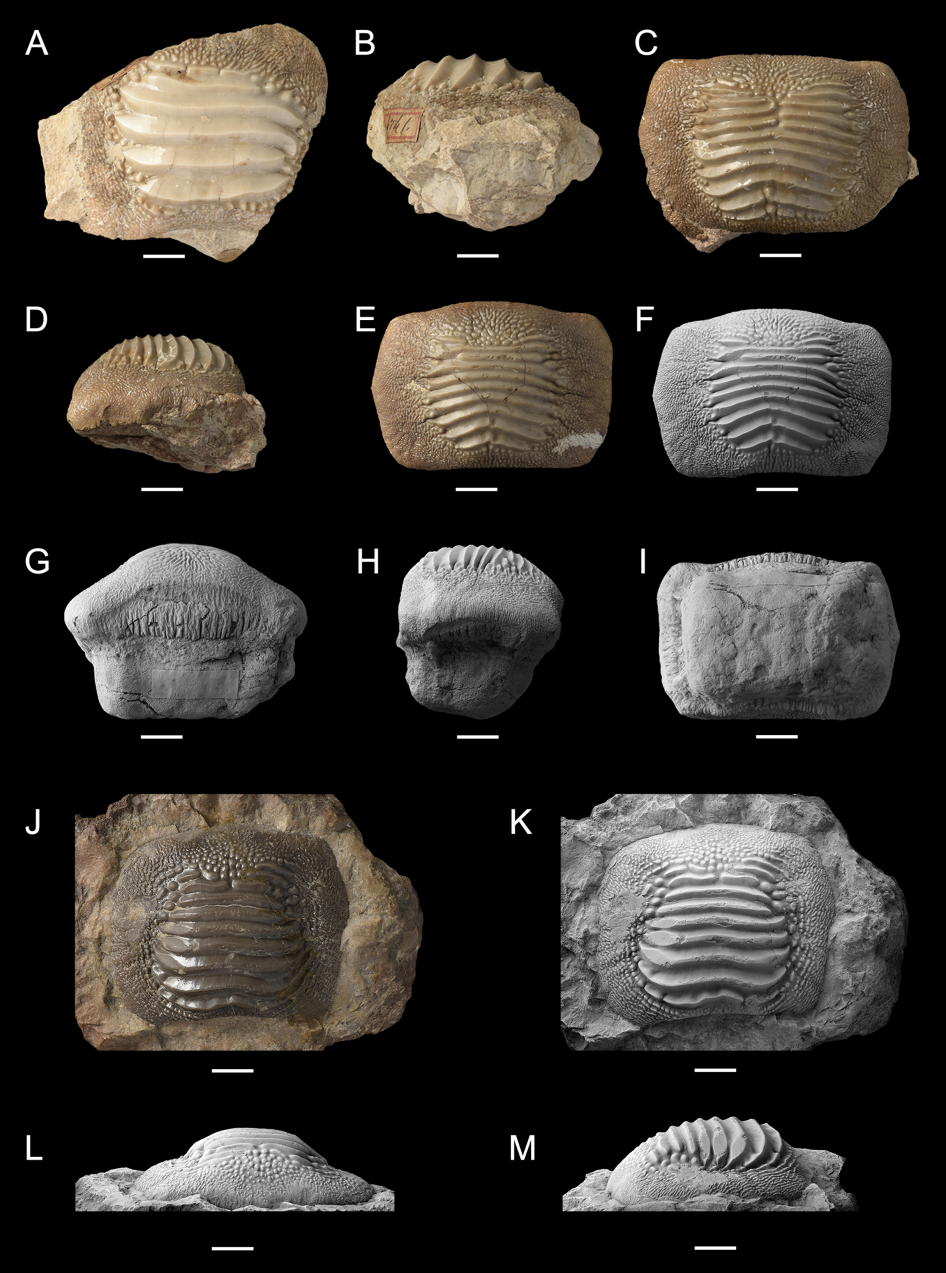
Isolated finds of *Ptychodus latissimus* Agassiz, 1835 from northeastern Italy in occlusal (A, C, E, F, J and K), anterior (G and L), lateral (B, D, H and M) and inferior (I) views. Specimens documented by color photos (A–E and J) and photos after smoking treatment (F–I and K–M). (A and B) Lateral tooth MGP-PD 6741. (C and D) Symphyseal tooth MGP-PD 12201. (E–I) Symphyseal tooth MGP-PD 12203. (J–M) Symphyseal tooth MGP-PD 27249. Scale bars equal 10 mm.

**Figure 11:**
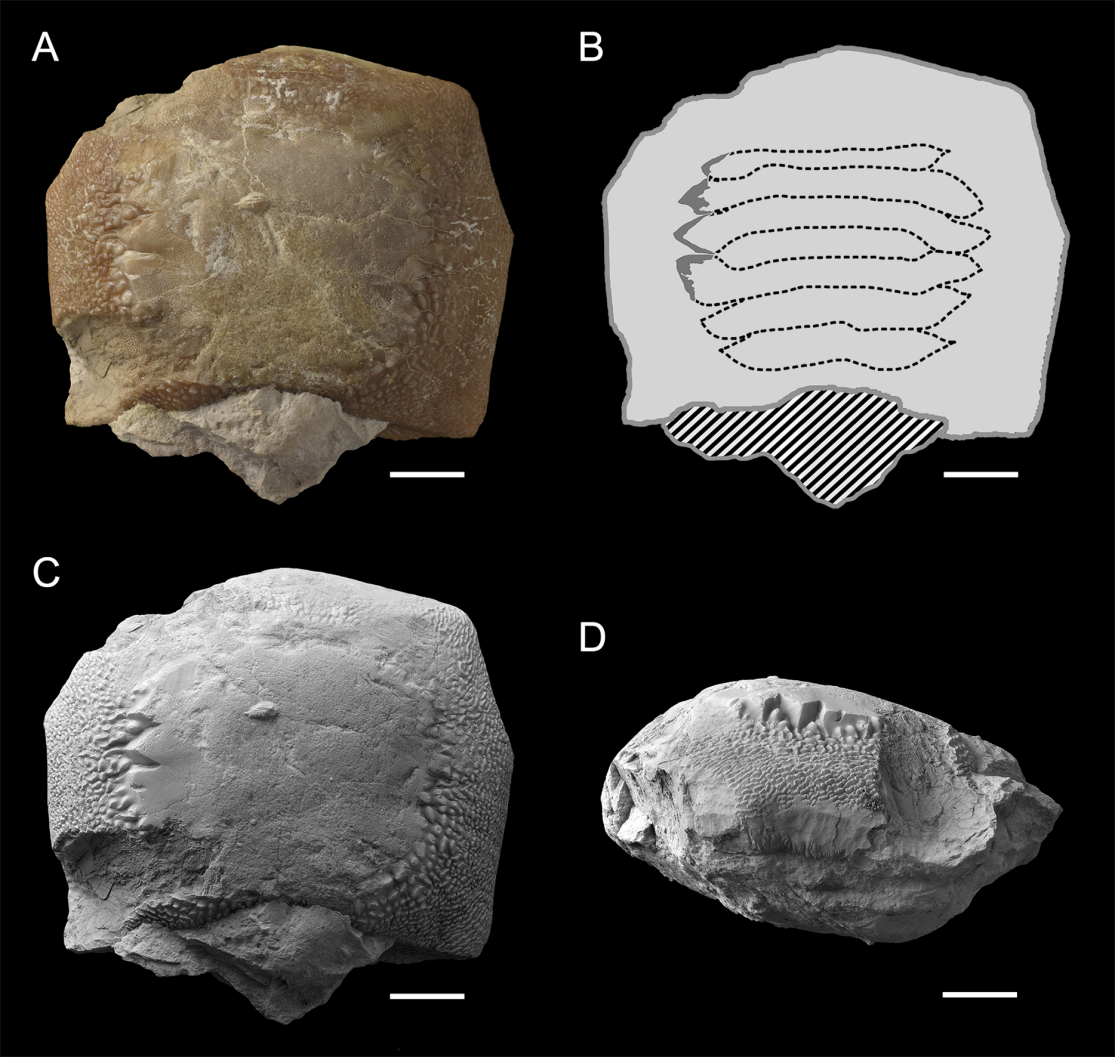
Worn tooth MGP-PD 6729 (A, B, C and D) of *Ptychodus latissimus* Agassiz, 1835 in occlusal (A and C) and lateral (D) views and interpretative drawing (B) of its reconstructed occlusal ornamentations. Striped pattern, matrix; light gray, dental crown; dark gray, preserved ornamentation, dotted line, reconstruction. Specimen documented by color photos (A) and photos after smoking treatment (C and D). Scale bars equal 10 mm.

**Figure 12:**
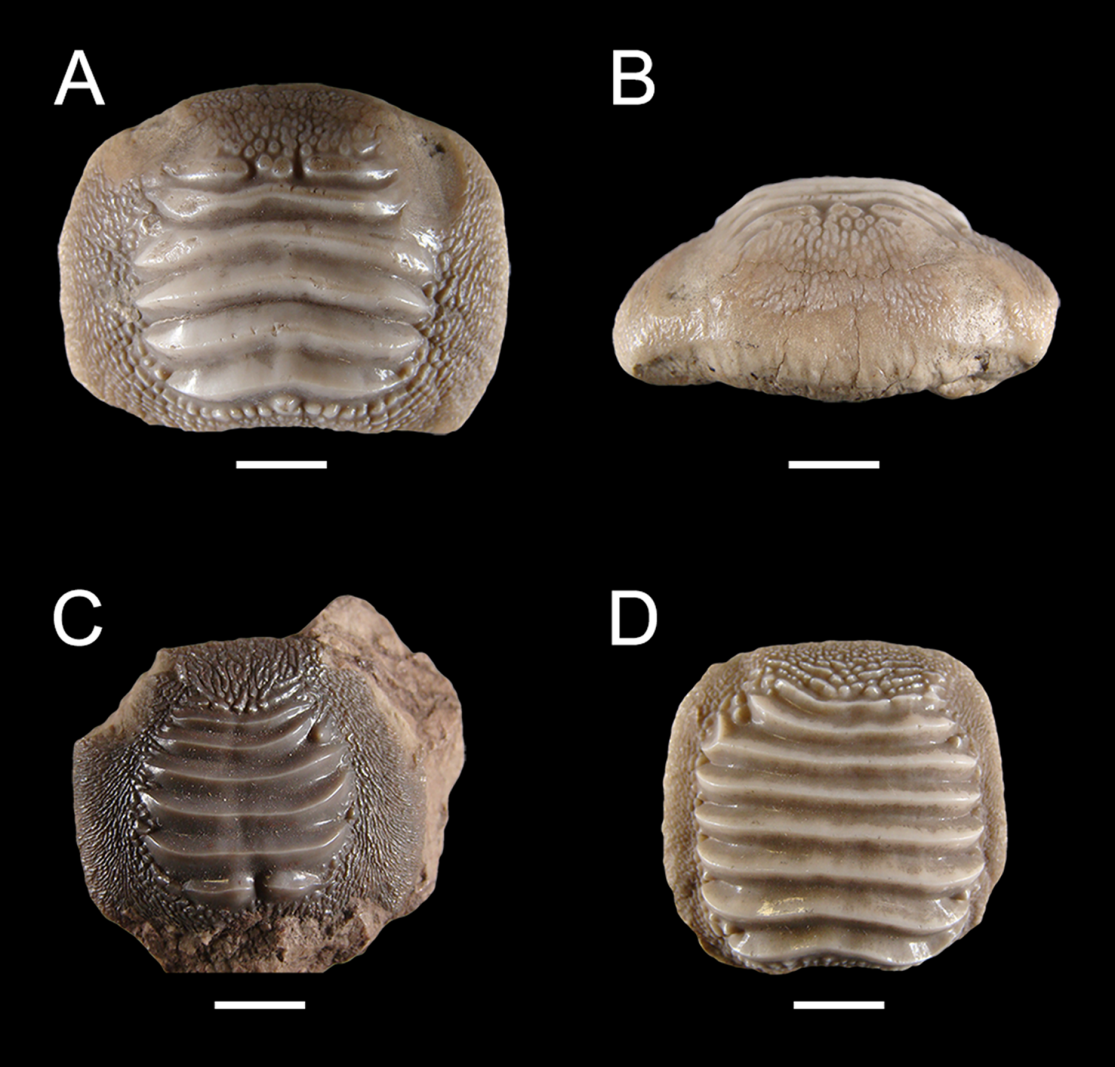
Isolated finds of *Ptychodus latissimus* Agassiz, 1835 from northeastern Italy in occlusal (A, C, D and K) and anterior (B and L) views. (A and B) Symphyseal tooth NHMW 8543α. (C) Symphyseal tooth NHMW 8543β. (D) Lateral tooth NHMW 8543γ. Scale bars equal 10 mm.

**Figure 13:**
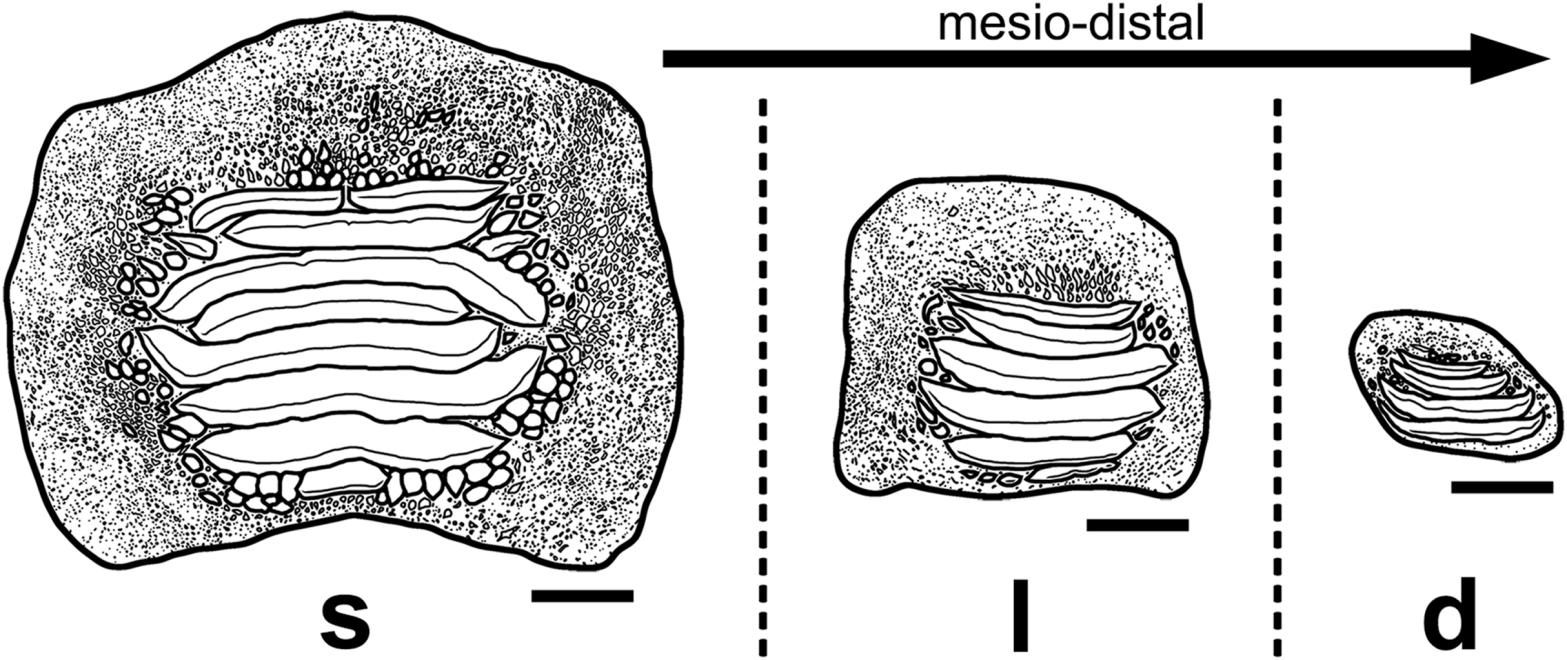
Interpretative drawings of the three main morphotypes (s, symphyseal; l, lateral; d, distal) within the lower dental plates of *Ptychodus latissimus* Agassiz, 1835 based on the associated specimen MCSNV v.1612 (see also Figs. 4–8). The morphological variants are figured in occlusal view and arranged according to their mesio-distal position in the dentition. Scale bars equal 10 mm.

**Figure 14:**
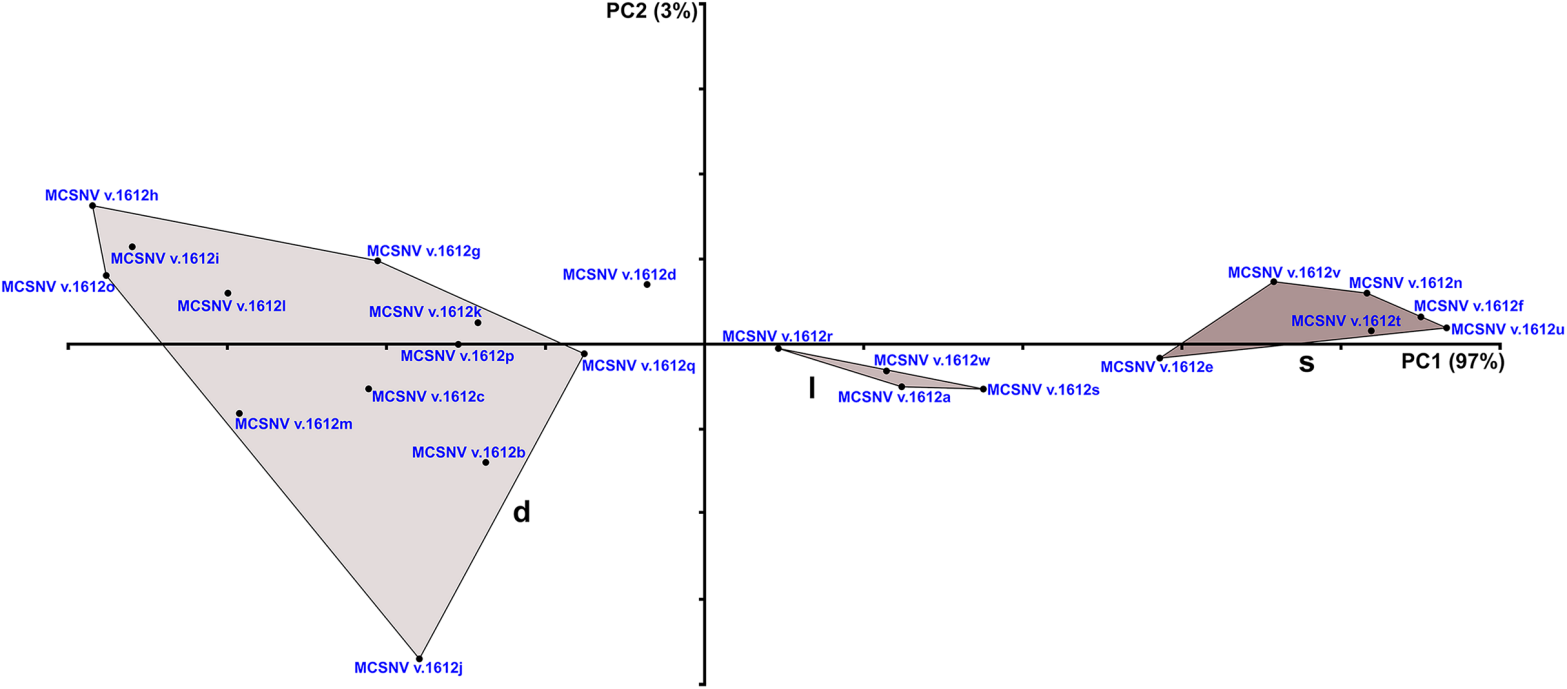
PCA plot of the data set from the specimen MCSNV v. 1612 (see Table 1; Table S2). Shades of gray (dark to light gray) are related to identification of the three morphotype groups in mesio-distal direction (s, symphyseal; l, lateral; d, distal). Scale of PC1 axis equals 0.1. Scale of PC2 axis equals 0.01.

(Selected synonyms)

“dens molaris piscis majoris marini”; Bruckmann (1737): 11-12, pl. 4, fig. 5.

“palati di pesce”; Brocchi (1814): 174.

“palati”; Catullo (1818): 20.

*Diodon*; Catullo (1820): 390, pl.7, fig. C.

“einer der hintern Kieferzähne eines grossen Fisches”; Von Schlotheim (1822): 70, pl. 13, figs. 2a–2c.

p. “Teeth allied to *Diodon*”; Mantell (1822): 231, pl. 32, fig. 19 (non figs. 17, 18, 20, 21, 23-25, 27 and 29).

p. *Diodon*; Catullo (1827): 149, pl. 3, fig. C (non figs. A and B).

“fossil fish tooth”; Von Sternberg (1827): 98, pl. 1, fig. 1.

“fish related to the Diodon”; Mantell (1833): 132, text fig. p. 133.

*Ptychodus Schlottheimii* (sic); Agassiz (1834): 69 (nomen oblitum).

† *Ptychodus latissimus* Ag.; Agassiz (1835): 54, Feuilleton additionnel.

*Ptychodus latissimus* Agass.; Agassiz (1837): pl. 25a, figs. 5 and 6 (non figs. 1–4, 7 and 8), atlas vol. III.

*Pt. latissimus* Agass.; Agassiz (1838): pl. 25b, figs. 24–26 atlas vol. III.

*Ptychodus latissimus* Ag.; Agassiz (1843): 156, vol. III (non syn.).

v *Ptychodus latissimus* Agass.; Pellegrini (1883): 145.

*Ptychodus latissimus* Agassiz; Bassani (1886): 145, pl. 9, fig. 11.

v *Ptychodus latissimus* Agass.; Nicolis (1889): 61, pl. 1.

*Ptychodus latissimus* Ag.; Sacco (1905): 255, pl. 8, figs. 11a–11c.

p. *Ptychodus latissimus* Agassiz; Woodward (1912): 235, text figs. 74 and 75, pl. 50, figs. 4, 6–8, 11 and 12 (non figs. 1–3, 5, 9, 10 and 13–16).

v *Pt. latissimus* Agass.; Canavari (1916): 67, pl. 12, fig. 17.

vp. *Ptychodus latissimus* Ag.; D’Erasmo (1922): 17, pl. 2, figs. 9, 10 and 12–14 (non figs. 11 and 15).

vp. *Ptychodus polygyrus* Ag.; D’Erasmo (1922): 20, pl. 2, fig. 16 (non figs. 17–20).

*Pt. latissimus* Ag.; Coggi (1964): 119, text fig. 1.

p. *Ptychodus paucisulcatus* Dixon F. 1850; Herman (1977): 67, pl. 2, fig. 10c (non figs. 10a and 10b) (non syn.).

v “pesci fossili simili alle razze”; Aspes & Zorzin (1984): 16, text fig. p. 16.

v *Ptychodus latissimus*; Zorzin (2001): 97, text fig. p. 97.

v *Ptychodus latissimus*; Zorzin & Vaccari (2005): 128, text-fig. p. 127.

*Ptychodus latissimus* Agassiz, 1835; Brignon (2015): 6.

*Ptychodus schlotheimii* Agassiz, 1834; Brignon (2015): 7, text-fig. 1.

*Ptychodus*; Zorzin (2017): text-fig. p. 68.

vp. *Ptychodus* cf. *latissimus*; Amadori et al. (2019a): 236, pl. III, figs. A and B^1^ (non fig. C).

*Ptychodus latissimus* Agassiz, 1835; Amadori et al. (2020): 4, fig. 3 (cum syn.).

***Type material***. The specimens from the Mantell collection housed in the NHMUK figured in Mantell (1822: pl. 32*)* and in Agassiz (1837); most of them come from the “Turonian Zone” of the Lewes Chalk Member (East Sussex, southern England; see also Woodward, 1889, 1912; Brignon, 2019; Amadori et al., 2020). Mantell (1822) did not provide detailed information about the original locality of the isolated teeth from Sussex figured in pl. 32 (see also Brignon, 2019). Amadori et al. (2020), who also emended the taxon, recently designated a lectotype of *Ptychodus latissimus* Agassiz, 1835 among the type specimens from the Lewes Chalk Member.

***Diagnosis***. A species of *Ptychodus* with almost quadratic dental tooth crowns, flattened or slightly bulged centrally and transversally crossed by very thick and sharp, but short ridges; ridges with little or no bending at their lateral extremities, terminating abruptly but never forming loops; crested area never reaching tooth edges; transition between marginal and crested area often characterized by large granules; marginal area well-developed and covered by fine granulations; granules sometimes extending to the ridge ends and curving anteriorly (see also Amadori et al., 2020).

***Referred material***. Two tooth sets (catalogue numbers MCR FO 00662, MCSNV v.1612) and 28 isolated specimens or cluster of teeth (catalogue numbers MCSNV v.12510, MCSNV v.12511, MCSNV v.12513, MCSNV v.12515, MCSNV v.12516, MCSNV v.12517, MCR FO 00663E, MGP-PD 3803, MGP-PD 3804, MGP-PD 6729, MGP-PD 6741, MGP-PD 6742, MGP-PD 7347, MGP-PD 8491, MGP-PD 8495, MGP-PD 12201, MGP-PD 12202, MGP-PD 12203, MGP-PD 14028, MGP-PD 14030, MG-PD 23538, MGP-PD 23540, MGP-PD 27249, MSNUP 272, MGC VR 47890, MCV 779 and NHMW 8543) showing different degrees of preservation.

***Occurrence & age***. Upper Cretaceous Scaglia Rossa Formation of northeastern Italy, “Lastame” lithofacies, Lessini Mountains, Verona and Vicenza provinces (MCSNV v.1612, MCSNV v.12510, MCSNV v.12515, MCSNV v.12516, MCSNV v.12517, MGC VR 4789, MGP-PD 6729, MGP-PD 6741, MGP-PD 6742 and MGP-PD 8495); “Pietra di Castellavazzo” lithofacies, Piave Valley, Belluno province (MGP-PD 3804, MGP-PD 23538, MGP-PD 23540, MGP-PD 27249, NHMW 8543).

The calcareous nannofossil content of the associated tooth set MCSNV v.1612 indicates the UC7-UC9 zones of Burnett (1999), corresponding to an early-late Turonian age (see Ogg & Hinnov, 2012), which is consistent with other “Lastame” fossils (see “Geological setting”). Matrix samples from MGP-PD 27249 and MGP-PD 7347 were almost devoid of calcareous nannoplankton and the amount of sedimentary rock was inadequate to analyze planktic foraminifera. There is no clear indication of the stratigraphic occurrence of several other specimens. Specimens MGP-PD 12201, MGP-PD 12202 and MGP-PD 12203, coming from the surroundings of Valdagno, Lessini Mountains (Vicenza province, Italy), show portions of matrix whose lithology resembles that of “Lastame”. Specimen MCV 779 probably comes from the surroundings of Novale (Vicenza province, Italy), together with two other almost identical teeth of the Dal Lago collection figured by D’Erasmo (1922: pl. II, figs. 12 and 13; see Figs. 1H and 1I*)*, which are lost.

The original localities of the Italian specimens examined herein are indicated in Fig. 3. Although details on the provenance of MCV 779, MCSNV v.12511, MCSNV v.12513, MGC VR 4789, MGP-PD 3803, MGP-PD 7347 and MSNUP 272 are currently unknown, the lithological composition of the embedding matrix suggests that MSNUP 272 comes from the “Lastame”, whereas MGP-PD 3803 and MGP-PD 7347 probably come from the “Pietra di Castellavazzo” (Belluno province). Specimen MCR FO 00662 comes from the “Lastame” cropping out near Mazzurega (Verona) and was probably donated to the Rovereto Museum by Gaetano Pellegrini in 1883 (Rasera, 2004). According to Rasera (2004), other specimens of *P. latissimus* coming from Vezzena (Trento), which is not far from Levico, were also donated to the Museum of Rovereto by Dario Graziadei in 1911. Five isolated teeth labeled as “*P. latissimus* from Levico” (MCR FO 00663A-E) are housed in the collections of the Rovereto Museum (see Amadori et al., 2019a). Four of them, however, represent indisputably *P. decurrens* Agassiz, 1838 (MCR FO 00663A-D) and are Cenomanian in age based on based on the calcareous nannofossil content of their matrix, whose lithology also suggests a provenance from the Scaglia Variegata Alpina Formation (Amadori et al., 2019a). Only the fifth tooth (MCR FO 00663E), here figured and described, is attributable to *P. latissimus*. Its state of preservation and associated reddish marly matrix indicate that the fossil likely comes from the Turonian-Maastrichtian Scaglia Rossa Formation. Unfortunately, the matrix of the tooth is insufficient even for calcareous nannofossil sampling, hampering a more accurate dating. Based on the available data, most of the specimens examined possibly come from Turonian beds of the “Lastame” lithofacies, whereas the exact stratigraphic position of some sparse teeth (including material from Castellavazzo) is still unknown or problematic.

***Description***. The associated tooth set MCR FO 00662 exhibits six teeth (labelled 662α, 662β, 662γ, 662δ, 662ε and 662ζ in Fig. 9M), which are all characterized by similar morphologies. The teeth have a rectangular crown crossed by three to seven thick ridges, which terminate abruptly not reaching the lateral tooth margins. Large bumps are scattered around the ends of the ridges. Occlusal crown abrasion, damage or matrix covering hampers a reliable estimation of the number of ridges. Tooth imprints and fragments of occlusal ridges are also recognizable on the surface of the embedding rock.

In occlusal view, the isolated tooth MCR FO 00663E (Figs. 9N–9Q) is characterized by a longitudinally developed rectangular crown bearing five transverse ridges restricted the central part of the tooth; coarse granulation and large bumps cover the lateral marginal areas. In posterior and anterior views (Figs. 9O and 9P), the dental crown displays bulgy central, crested areas, while both the lateral margins are distally tilted. The root is almost completely absent.

The associated tooth set MCSNV v.1612 (Figs. 2 and 4–8) comprises about 52 teeth, embedded in a slab of nodular limestone, and additional impressions of other teeth, which were detached from the matrix and subsequently lost (see Figs. 4 and 5). The teeth exhibit different degrees of completeness, from entire dental crowns to tooth fragments; however, no roots are preserved or are exposed from the matrix. The occlusal ornamentation of the teeth in MCSNV v.1612 (see Fig. 4) is very similar to each other, while their size and general shape exhibit a considerable variation. Three different morphotypes, here labeled “s” (symphyseal), “l” (lateral) and “d” (distal), are recognizable in MCSNV v.1612 (see Fig. 4). Morphotype “s” (Fig. 13s) is represented by the largest teeth on the slab (e.g., MCSNV v.1612e, MCSNV v.1612f, MCSNV v.1612n, MCSNV v.1612t, MCSNV v.1612u, MCSNV v.1612v in Fig. 5) and consists of a symmetrical, rectangular crown crossed by seven to eight thick, sharp ridges (e.g., Fig. 8). The ridges curve slightly at their ends, which are often surrounded by large bumps. Coarse granulations cover the entire marginal area. Some of these large teeth (e.g., MCSNV v.1612e in Fig. 5) included in the morphotype “s” are markedly abraded (see Fig. 6).

Morphotype “l” (e.g., MCSNV v.1612a, MCSNV v.1612r, MCSNV v.1612s, MCSNV v.1612w in Fig. 5) exhibits an asymmetrical outline with an almost rectilinear mesial and a curved distal edge. Five to six thick ridges characterize the occlusal surface (see Fig. 8).

Morphotype “d” includes the smallest teeth (e.g., MCSNV v.1612g, MCSNV v.1612h, MCSNV v.1612i, MCSNV v.1612j, MCSNV v.1612k, MCSNV v.1612l, MCSNV v.1612m, MCSNV v.1612o, MCSNV v.1612p, MCSNV v.1612q in Fig. 5) exhibiting a markedly asymmetric crown with four sharp, occlusal ridges (see Fig. 7). The tooth outline has anterior and distal edges merged in a unique convex curve, while the mesial side is convex or distally tilted. The posterior sulcus is shallow. A summary of the identified morphotypes is provided in Table 1.

**Table 1:** Summary of the morphotypes (Mpt) identified herein based on the associated specimen MCSNV v.1612 and related to the interpretation of the tooth position (ITP) within the lower dental plate.

Catalogue number	Mpt	ITP
MCSNV v.1612a	l	Lateral
MCSNV v.1612b	?	?
MCSNV v.1612c	?	?
MCSNV v.1612d	?	?
MCSNV v.1612e	s	Symphyseal
MCSNV v.1612f	s	Symphyseal
MCSNV v.1612g	d	Distal
MCSNV v.1612h	d	Distal
MCSNV v.1612i	d	Distal
MCSNV v.1612j	d	Distal
MCSNV v.1612k	d	Distal
MCSNV v.1612l	d	Distal
MCSNV v.1612m	d	Distal
MCSNV v.1612n	s	Symphyseal
MCSNV v.1612o	d	Distal
MCSNV v.1612p	d	Distal
MCSNV v.1612q	d	Distal
MCSNV v.1612r	l	Lateral
MCSNV v.1612s	l	Lateral
MCSNV v.1612t	s	Symphyseal
MCSNV v.1612u	s	Symphyseal
MCSNV v.1612v	s	Symphyseal
MCSNV v.1612w	l	Lateral

**Note:**

See Fig. 5 for the placement of the teeth on the slab MCSNV v.1612.

The isolated tooth MCSNV v.12510 (Figs. 9A and 9B) lacks the root and most of the marginal area; nevertheless, its occlusal ornamentations are identical to those of MCSNV v.1612t and MCSNV v.1612u (Figs. 5 and 8). Specimen MCSNV v.12511 includes five tooth fragments with only little portions of the thick occlusal ridges being preserved. Specimen MCSNV v.12513 consists of two teeth (labelled herein “MCSNV v.12513α” and “MCSNV v.12513β”). Specimen MCSNV v.12513α (Figs. 9C and 9D) has a quadratic and slightly asymmetric crown. Seven thick, sharp ridges cross the occlusal surface and terminate abruptly, not reaching the lateral tooth edges. No traces of wear are recognizable, but whitish furrows cover the rearmost ridges (see Fig. 9C). The marginal area is characterized by a coarse granulation and large bumps around the ends of the ridges. In lateral view (Fig. 9D), the thick, flat crown protrudes on a thick, squared root. The posterior side of the root is perpendicular to the base of the crown, whereas the anterior one tilts posteriorly. Specimen MCSNV v.12513β includes only the crested area with six thick ridges, which terminate abruptly. MCSNV v.12515 consists of three teeth (labelled herein “MCSNV v.12515α”, “MCSNV v.12515β” and “MCSNV v.12515γ”). Specimen MCSNV v.12515α is a small tooth with a crown characterized by an irregular, asymmetric outline and six thin ridges; the crested and marginal area are clearly separate from each other. The dental morphologies of specimens MCSNV v.12515β, MCSNV v.12515γ and MCSNV v.12517 are consistent with those observed in MCSNV v.12513α (see Figs. 9C and 9D); they differ from the latter by having five to six ridges and damaged crowns along the edges. At the center of the crested area of MCSNV v.12515γ, slight traces of wear are recognizable.

Specimen MCSNV v.12516 includes two detached teeth (labelled herein “MCSNV v.12516α” and “MCSNV v.12516β”). MCSNV v.12516α (Figs. 9E–9H) exhibiting a rectangular crown crossed by seven thick, sharp ridges and a marginal area covered by coarse granulations. The posterior sulcus and anterior protuberance are poorly developed. In occlusal view (Fig. 9E), most of the ridges are abraded at their lateral ends, which are surrounded by large bumps. In lateral view (Fig. 9H), the crested area in the center of the crown is gently raised. Specimen MCSNV v.12516β displays only the right half of its crown with occlusal ornamentation, including six ridges, similar to that exhibited by MCSNV v.12516α. Although specimen MCV 779 lacks the right edge, it is almost identical to MCSNV v.12516α (Figs. 9E–9H) and no traces of wear are recognizable.

The small teeth MGC VR 47890 (Figs. 9R and 9S), MGP-PD 8495 and MGP-PD 14030 are similar to MCSNV v.12513 (Figs. 9C and 9D) in their general morphology and ornamentations (see above), but none of them has the root preserved. In occlusal view, the ridges of MGC VR 47890 and MGP-PD 8495 are markedly abraded at their ends (e.g., Fig. 9R). In lateral view, the tooth crowns of MGC VR 47890, MGP-PD 8495 and MGP-PD 14030 have a bulgy outline (e.g., Fig. 9S). Specimens MGP-PD 3803, MGP-PD 3804, MGP-PD 6742, MGP-PD 8491, MGP-PD 14028 and MGP-PD 23540 are fragmentary teeth with markedly crested areas surrounded by dark, reddish matrix. The poor preservation of most of these teeth does not allow determining the correct number of occlusal ridges; at least five are recognizable. Weak traces of wear are observable on the ridges of specimens MGP-PD 3804, MGP-PD 6742, MGP-PD 8491 and MGP-PD 14028. Specimen MGP-PD 23538 includes four tooth fragments morphologically similar to each other and characterized by four to six parallel ridges; two of these fragmentary teeth exhibit feeble abrasions on the crested area. Specimen MGP-PD 6729 (Fig. 11) is a large, rectangular tooth with symmetrical outlines and occlusal ridges that are completely abraded, except at their marginal extremities. These ridge ends are thick and clearly distinct from the marginal area. The marginal ornamentations seem to be quite similar to those of MCSNV v.12510 (Figs. 9A and 9B; see above). Specimen MGP-PD 6741 (Figs. 10A and 10B) has a badly preserved crown that is crossed by four thick, sharp ridges, which are restricted to the center of the tooth; two additional thin ridges are placed anteriorly (see also Fig. 1E). Although most of the marginal area is broken, the occlusal ornamentation seems to be asymmetrical. The ridges do not reach the tooth margins and they are surrounded by large bumps. In lateral view (Fig. 10B), the crown is massive and bulged, while the root is not preserved. Specimens MGP-PD 12201 (Figs. 10C and 10D), MGP-PD 12202 (Fig. 1F) and MGP-PD 12203 (Figs. 10E–10I) have a rectangular and symmetrical outline. Ten to eleven parallel, thick ridges, surrounded by large bumps, cross the occlusal surface. The anteriormost ridges are often thin and disrupted (e.g., Figs. 10C, 10E and 10F). The crested area is clearly distinct from the marginal one, which is covered by coarse granulation. In lateral view (e.g., Figs. 10D and 10H) the crown is massive and bulged. Only specimens MGP-PD 12202 (Fig. 1F) and MGP-PD 12203 (Figs. 10E–10I) have rectangular and massive roots with a shallow antero-posterior sulcus (e.g., Figs. 10I–10G). Whitish and irregular furrows are scattered on the occlusal surface of specimen MGP-PD 12201 (Figs. 10C and 10D). The anterior portion of the crown of specimen MGP-PD 12202 is broken on its left side (see Fig. 1F).

Specimens MGP-PD 7347, MGP-PD 27249 (Figs. 1D and 10J–10M) and MSNUP 272 (Figs. 9I–9L) exhibit seven to twelve occlusal ridges, with curved lateral edges. These three specimens are similar to MGP-PD 12201 (Fig. 10C), MGP-PD 12202 (Fig. 1F) and MGP-PD 12203 (Fig. 10E) in their general outline and occlusal ornamentation (see above). Specimen MSNUP 272 (Figs. 9I–9L) exhibits a rectangular, poorly preserved root, while the inferior portion of MGP-PD 7347 and MGP-PD 27249 (Figs. 10J–10M) are embedded in a reddish matrix. Marked traces of wear are observed on the left side of the crown of MGP-PD 27249 (Figs. 10J and 10K). These abrasions affect the ends of the ridges on both sides and part of the marginal granulations (see also Fig. 10M). Conversely, the abrasions on MGP-PD 7347 are barely recognizable on the occlusal ridges due to their poor preservation. Specimen NHMW 8543 (Fig. 12) includes three isolated teeth (labelled herein NHMW 8543α, NHMW 8543β and NHMW 8543γ) with well-preserved crowns but missing roots. The general shape and occlusal ornamentation of NHMW 8543α (Figs. 12A and 12B) and NHMW 8543β (Fig. 12C) are consistent with those of MCSNV v.12516α (Figs. 9E–9H; see above). Moreover, NHMW 8543α (Figs. 12A and 12B) shows marked abrasions on most of its anterior occlusal surface. Specimen NHMW 8543γ (Fig. 12D) have a quadratic crown crossed by eight thick ridges and is morphologically similar to MCSNV v.12513α (Figs. 9C and 9D; see above). Measurements and other morphological details are provided in Tables 2 and 3 (Supplementary Material).

***Remarks***. Only two associated tooth sets of *Ptychodus latissimus* are known so far. 85 teeth (MGL 2021-2022) were originally found associated within a single slab in the surroundings of Condé (northern France). Later, the teeth were detached from the matrix by Leriche (1906: pl. 5, figs. 1 and 2*)* to provide an interpretative reconstruction of the upper and lower dental plates of this low-crowned species (see also Leriche, 1902; Woodward, 1912). This material is currently housed in the Musée d’Histoire Naturelle de Lille (see Malvesy et al., 2002). Another associated specimen (BMM 007333) comes from southern England and is housed in the Booth Museum of Brighton (see Hamm, 2020: fig. 69). Specimens MCR FO 00662 and MCSNV v.1612 that are described herein increase the number of known associated specimens attributed to *P. latissimus* from two to four. The articulated dental plates of *Ptychodus* studied here show morphological characters, such as a mesially placed bilateral symmetry of the teeth and the mesio-distal decrease in tooth size, which are shared by both cuspidate and non-cuspidate species (e.g., *P. decurrens*, *P. mammillaris*, *P. mediterraneus* and *P. mortoni*; Woodward, 1912; Shimada, 2012; Amadori et al., 2020; Hamm, 2017, 2020). Assuming that these features occur also in species for which articulated finds are still unknown (e.g., *P. latissimus*), the original tooth arrangement of isolated finds or associated specimens with displaced teeth can be determined or at least supposed (see also Hamm, 2017; Amadori et al., 2019a, 2019b). The size and general morphology (e.g., slightly bulged crown) of all the teeth preserved in MCSNV v.1612 (Fig. 4) concur to suggest an attribution to a lower dentition of an individual of *Ptychodus latissimus* Agassiz, 1835. In particular, the large symmetrical teeth (e.g., MCSNV v.1612e, MCSNV v.1612f, MCSNV v.1612n, MCSNV v.1612t, MCSNV v.1612u, MCSNV v.1612v in Figs. 5 and 8) assigned to the morphotype “s” (s in Fig. 13) were probably arranged into a symphyseal row of the dental plate. The teeth identified as morphotype “l” (l in Fig. 13) in MCSNV v.1612 (e.g., MCSNV v.1612s in Figs. 5 and 8) are lateral teeth originally placed within the lateral rows between symphyseal and the distal teeth, while the small teeth (e.g., MCSNV v.1612g, MCSNV v.1612h, MCSNV v.1612i, MCSNV v.1612j, MCSNV v.1612k, MCSNV v.1612l, MCSNV v.1612m, MCSNV v.1612o in Figs. 5 and 7) attributed to the morphotype “d” (d in Fig. 13) were placed within the distalmost rows of the tooth plate.

The presence of a further morphotype cannot be excluded based on the occurrence of three small teeth (e.g., MCSNV v.1612b, MCSNV v.1612c, MCSNV v.1612d; see Fig. 5), which seem to have squared and bulgy crowns crossed by five ridges. Unfortunately, the identification of this fourth morphological variant remains uncertain, as the edges of these three dental elements are scarcely exposed and difficult to be prepared (see Fig. 4). The largest tooth (662-α in Fig. 9M) of set MCR FO 00662 is interpreted herein as a lower symphyseal, while the others (662-β to 662-ζ in Fig. 9M) can be considered as lateral teeth originally placed close to the symphyseal row (see also Amadori et al., 2019a). We tested morphometric measurements (crown width and crown length) of tooth set MCSNV v. 1612 (see Table 1 for morphotype identification and Table S2 for measurements) with a PCA analysis to preliminarily investigate this variability from a quantitative perspective and to provide further support for the validity of qualitative identifications of the morphotypes. Positive values of PC1 are related to large size in both CW and CL. The positive values of PC2, on the other hand, are related to the CW, whereas negative values are related to CL (see “Loadings plot” in Supplemental Data). The PCA analysis (Fig. 14) evidenced three distinct groups that reflect the three morphotypes described above, not representing artificial groupings and thus corroborating the qualitative morphological identification within the associated tooth set MCSNV v. 1612.

Isolated specimens include teeth from both lower and upper dentitions. Teeth interpreted herein as coming from the lower dentition are MCV 779, MCSNV v.12510, MCSNV v.12515α, MCSNV v.12516α, MGC VR 47890, MGP-PD 6729, MGP-PD 6741, MGP-PD 7347, MGP-PD 8495, MGP-PD 12201, MGP-PD 12202, MGP-PD 12203, MGP-PD 14030, MGP-PD 27249, MSNUP 272, NHMW 8543α and NHMW 8543β. In particular, MCV 779, MCSNV v.12510 (Figs. 9A and 9B), MCSNV v.12516α (Figs. 9E–9H), MGP-PD 7347, MGP-PD 12201 (Figs. 10C and 10D), MGP-PD 12202 (Fig. 1F), MGP-PD 12203 (Figs. 10E–10I), MGP-PD 27249 (Figs. 10J–10M), MSNUP 272 (Figs. 9I–9L), NHMW 8543α (Figs. 13A and 13B) and NHMW 8543β (Fig. 13C) are recognized as symphyseal teeth. The transition between the preserved ridge ends in MGP-PD 6729 (Fig. 11) and its marginal area is not gradual. Furthermore, the interpretative reconstruction proposed herein (Fig. 11B) exhibits thick ridges terminating abruptly and limited to the center of the dental crown. Based on the reconstructed ornamentation pattern, together with the size and the shape of the crown (see “Description”, above), MGP-PD 6729 is interpreted as a symphyseal tooth. The almost quadratic teeth MGC VR 47890 (Figs. 9R and 9S), MGP-PD 8495, MGP-PD 14030 were probably originally arranged laterally to the symphyseal row. Based on its rectangular and asymmetrical shape, tooth MGP-PD 6741 probably was placed slightly more distally within the right portion of the dentition. Specimen MCSNV v.12515α shares some features with those assigned to the morphotype “d” (Fig. 13d) in MCSNV v.1612 (e.g., Fig. 7), and thus referred to one of the distalmost rows of the dental plate.

Teeth referred herein to the upper dental plate are MCSNV v.12513α (Figs. 9C and 9D), MCSNV v.12515β, MCSNV v.12515γ, MCSNV v.12517, NHMW 8543γ (Fig. 12D). These are lateral teeth probably belonging to indeterminate rows of the left portions of the dentition. Unfortunately, MCSNV v.12511, MCSNV v.12513β, MCSNV v.12516β, MGP-PD 3803, MGP-PD 3804, MGP-PD 6742, MGP-PD 14028, MGP-PD 23538 and MGP-PD 23540 are too fragmentary for assignment to any specific row within the dental plates. Slight morphological qualitative variations (e.g., curvature at the ends of the ridges) observed among teeth assigned to the same position within the crushing plates provide evidence of intraspecific variability for this low-crowned taxon. However, the general morphology of isolated teeth supports the identification of the morphotypes described herein for *P. latissimus*.

Whitish and irregular furrows, similar to those described herein on the occlusal ornamentation of MCSNV v.12513α (Figs. 9C and 9D) and MGP-PD 12201 (Figs. 10C and 10D), have been recently observed on the enameled ridges of other low-crowned taxa of *Ptychodus* (e.g., *P. mediterraneus*) and interpreted as the result of bioerosion by endolithic organisms (e.g., *Mycelites ossifragus* Roux, 1887) based on its unbranched patterns (see also Underwood, Mitchell & Veltkamp, 1999; Amadori et al., 2020).

## Discussion

### Comparisons

*Ptychodus latissimus* Agassiz, 1835 is commonly considered an iconic and well recognizable species usually identified based on few peculiar features, such as quadratic crowns and thick, sharp ridges (Agassiz, 1843; Woodward, 1912). Recently, ridge patterns, transition between crested/marginal area and marginal ornamentations have been recognized as the most taxonomically distinctive features for discriminating various low-crowned taxa, such as *P. latissimus* Agassiz, 1835, *P. marginalis* Agassiz, 1843, *P. martini* Williston, 1900a, *P. mediterraneus* Canavari, 1916 and *P. polygyrus* Agassiz, 1835 (see Amadori et al., 2020).

Another species that shares several characters with *Ptychodus latissimus* is *P. paucisulcatus* Dixon, 1850, as previously remarked by various authors (Woodward, 1912; Herman, 1977; Amadori et al., 2020; Hamm, 2020). Although these two taxa have been considered separate species for a long time, their mutual relationships remain uncertain (Woodward, 1887, 1912; Leriche, 1902, 1906, 1929; Herman, 1977; Antunes & Cappetta, 2002; Amadori et al., 2020; Hamm, 2020). Dixon (1850) introduced the species *P. paucisulcatus* based on a flat, isolated tooth and other still undescribed specimens. A still surviving syntype of *P. paucisulcatus* currently housed in Natural History Museum, London (NHMUK PV OR 25826; see Dixon, 1850: t. XXX, fig. 3), probably comes from the Turonian-Santonian of South Downs (UK) (Woodward, 1912; Friedman et al., 2016). Dixon (1850: 363*)* described ridges “…larger and stronger than in *P. latissimus* or any other species…” for this taxon. Dixon (1850) also reported a still unidentified specimen preserving 147 teeth, all morphologically consistent with NHMUK PV OR 25826. Later, Woodward (1912: 235*)* considered *P. paucisulcatus* Dixon, 1850 a junior synonym of *P. latissimus* Agassiz, 1835. In contrast with the Woodward interpretation, Herman (1977) and Antunes & Cappetta (2002) documented isolated teeth from the Upper Cretaceous of central-western Belgium and northwestern Angola, respectively, referring them to *P. paucisulcatus* Dixon, 1850.

The syntype NHMUK PV OR 25826 (see Dixon, 1850: t. XXX, fig. 3) of *Ptychodus paucisulcatus* Dixon, 1850 exhibits most of the typical dental features of *P. latissimus* Agassiz, 1835 recently highlighted by Amadori et al. (2020) based on a critical reassessment of the type series of Agassiz’s species. Nevertheless, the study of a larger sample would be mandatory for an accurate comparison and correct interpretation of the morphological differences between the two taxa. Therefore, additional studies and analyses based on a wider and more representative sample will be performed in the future in order to conclusively define their systematic affinities.

### Trophic specialization

Elasmobranchs independently evolved durophagy multiple times during their complex evolutionary history (see Summers, Ketcham & Rowe, 2004; Amadori et al., 2020). Batoids (stingrays, skates, sawfishes and guitarfishes) represent certainly one of the most successful groups of “hard-prey specialists” with various degrees of durophagous specializations (Summers, Ketcham & Rowe, 2004; Cappetta, 2012). Among selachimorphs, numerous species of horn sharks (Heterodontiformes) exhibit adductor muscles and postero-lateral molariform teeth suitable to crush hard-shelled prey (see Summers, Ketcham & Rowe, 2004; Cappetta, 2012). In addition, a slight degree of “grinding ability” is attributed to the tooth morphology of undetermined *Chiloscyllium* species (Orectolobiformes) from the Maastrichtian of North Africa (Cappetta et al., 2014). Molariform teeth included in dentitions with various degrees of heterodonty represent one of the most striking features of durophagous sharks (Summers, Ketcham & Rowe, 2004; Motta & Huber, 2012; Kolmann et al., 2015; Amadori et al., 2020). These polygonal and relatively flat teeth are often closely interlocked with each other, forming “pavement-like” dentitions suitable for crushing and grinding shelled prey (Woodward, 1912; Summers, 2000; Ramsay & Wilga, 2007; Shimada, Rigsby & Kim, 2009; Cappetta, 2012; Kolmann et al., 2015; Hamm, 2020). The degree of abrasion also indicates durophagous feeding in Mesozoic elasmobranchs (Claeson et al., 2010; Amadori et al., 2020). Isolated or associated molariform teeth, often abraded on their occlusal surface, are commonly documented in both low-crowned and high-crowned *Ptychodus* species suggesting specializations for preying on different types of shelled invertebrates (Woodward, 1912; Shimada, Rigsby & Kim, 2009; Cappetta, 2012; Shimada, 2012; Diedrich, 2013; Amadori et al., 2019a, 2019b, 2020; Amalfitano et al., 2020; Hamm, 2020).

In extant marine communities, sharks are commonly considered upper trophic level predators (Cortés, 1999; Jacobsen & Bennett, 2013). The trophic level (TL) and feeding preferences was inferred for various selachimorph groups based on measurements of stable isotopes, such as nitrogen and carbon in their tissues, and/or on their stomach contents (Cortés, 1999). Cortés (1999) concluded that most of the shark groups are third-level consumers (TL > 4). Orectolobiforms (TL = 3.6) and heterodontiforms (TL = 3.2) represent the main exceptions (second-level predators; see Cortés, 1999). Later, various authors expanded this estimate of the trophic level to various batoid groups (see Bizzarro, 2005; Ebert & Bizzarro, 2007; Belleggia et al., 2008; Woodland, Secor & Wedge, 2011; Jacobsen & Bennett, 2013; Coasaca-Céspedes et al., 2018; Rastgoo, Navarro & Valinassab, 2018; Yemışken et al., 2018). Batoids are mostly second-level predators, while the rare exceptions reaching a higher trophic level (TL > 4) manly belong to hypnids, gymnurids and torpedinids (see Bizzarro, 2005; Ebert & Bizzarro, 2007; Belleggia et al., 2008; Woodland, Secor & Wedge, 2011; Jacobsen & Bennett, 2013; Rastgoo, Navarro & Valinassab, 2018; Yemışken et al., 2018). In particular, the diet of the main extant durophagous elasmobranch groups (e.g., heterodontid sharks and stingrays) primarily consists of decapod crustaceans (e.g., shrimps and crabs; TL = 2.52), polychaetes (TL = 2.6) and mollusks excluding cephalopods (TL = 2.1; see Cortés, 1999; Jacobsen & Bennett, 2013). Upper level batoid predators belonging to hypnids, gymnurids and torpedinids mainly prey on bony fishes (TL = 3.24) and demersal/pelagic cephalopods (TL = 3.2), such as octopodids and squids, rather than decapods and mollusks. However, ginglymostomids and adults of some heterodontid taxa also might have similar dietary preferences (see Cortés, 1999; Jacobsen & Bennett, 2013; Powter, Gladstone & Platell, 2010).

The durophagous adaptations described herein based on the molariform teeth of *Ptychodus latissimus* Agassiz, 1835 are certainly more pronounced than in most other species of *Ptychodus*. Relatively flat crowns and occlusal sharp and thick ridges indicate a high adaptation for crushing hard-shelled prey in *P. latissimus*. This interpretation is additionally entrenched by worn teeth of this taxon exhibiting crested areas that are often completely abraded (e.g., Figs. 9M, 11A and 11C). Based on European and North American occurrences, various species of *Ptychodus*, including the low-crowned *P. latissimus*, probably occupied habitats with abundant shelled prey, such as inoceramid bivalves (see Williston, 1897, 1900a, 1900b; Hattin, 1988; Hamm & Shimada, 2004; Diedrich, 2013; Everhart, 2018; Hamm, 2020). Several species of inoceramid bivalves were documented from the Upper Cretaceous Scaglia Rossa of the Trentino and Veneto regions in northeastern Italy by poorly preserved material (Airaghi, 1904, 1931; Dhondt & Dieni, 1996). In particular, inoceramid bivalves are commonly reported from the “Lastame” lithozone (Scaglia Rossa Fm) of northeastern Italy (Cigala-Fulgosi et al., 1980; Astolfi & Colombara, 2003). Diedrich (2013) speculated that *P. latissimus* might have been able to feed on thick-shelled species of *Inoceramus* using its sculptured teeth with sharp and well-developed ridges to crack the resistant shells effectively. Nevertheless, a direct prey-predator relationship between inoceramids, or other shelled prey (e.g., ostreids), and *Ptychodus* remains elusive, exclusively supported by indirect evidences (Kauffman, 1972; Stewart, 1988; Ozanne & Harries, 2002; Diedrich, 2013; Everhart, 2017; Amadori et al., 2020; this study). Hamm (2020) recently suggested that the diet of *Ptychodus* could have been largely composed of benthic and nektonic ammonites. Many taxa of these shelled cephalopods (e.g., *Acanthoceras*, *Mammites* and *Sciponoceras*) indeed had broad geographic distributions similar to that of *Ptychodus* within the Western Interior Sea of North America (Hamm, 2020). Ammonites, including both evolute and involute forms, are also commonly found in both “Lastame” and “Pietra di Castellavazzo” lithofacies from the Upper Cretaceous of northeastern Italy (see also Amalfitano et al., 2017a, 2017b, 2017c, 2019a; Amadori et al., 2019b). Furthermore, the morphological variability, including various strongly ornamented taxa (e.g., *Acanthoceras* and *Mammites*), exhibited by North American ammonites could also indicate defensive adaptations against predators, such as low-crowned *Ptychodus* spp. (e.g., *P. latissimus* and *P. marginalis*; see also Hamm, 2020). Fossa-Mancini (1921) also noted that the ratio between “useful weight” (soft tissues) and “harmful weight” (skeleton) could be greater in ammonites than in other mollusks. This author thus concluded that these shelled cephalopods could ensure sufficient food even for individuals of *Ptychodus* reaching considerable body sizes (see also Shimada et al., 2010; Jambura & Kriwet, 2020). Like their extinct relatives (e.g., ammonites), living cephalopods have important and diversified roles in marine ecosystems, ranging from occasional scavengers (e.g., nautiloids) at lower trophic levels to carnivorous predators (e.g., squids; Olóriz & Villaseñor, 2010; Lukeneder, 2015). However, adults of modern nautiloids (e.g., *Nautilus pompilius*) exhibit a heterogeneous diet including both second- and third- level consumers (e.g., crustaceans, annelids and fishes) and they can also occupy upper trophic levels at maturity (TL = ~ 4; see Saisho & Tanabe, 1985; Kashiyama et al., 2010; Lukeneder, 2015). Preliminary attempts to analyze nitrogen isotopes in Albian nautiloids (e.g., *Cymatoceras*) provided uncertain and doubtful results exhibiting an opposite feeding trend compared to that of extant nautioids. Nevertheless, reliable estimates of the trophic level for both fossil nautiloids and ammonoids are not yet available (Kashiyama et al., 2010; Lukeneder, 2015). Feeding preferences primarily based on shelled prey (e.g., mollusks and decapods) suggest to considering *Ptychodus* as second-level consumer, like numerous extant durophagous elasmobranchs (e.g., heterodontid sharks and stingrays; see above). This could be assumed mainly for cuspidate species (e.g., *P. altior*, *P. occidentalis*), which would include crustaceans in their diet (see Shimada, Rigsby & Kim, 2009; Amadori et al., 2019b). Potentially able to crush both bivalves (e.g., inoceramids) and cephalopods (e.g., ammonites) even protected by thick and horned shells, *Ptychodus latissimus* and other low-crowned taxa probably occupied higher, important roles in the trophic web as third-level predators.

## Conclusions

The Italian record of *Ptychodus latissimus* Agassiz, 1835 is primarily represented by numerous isolated teeth and rare associated finds mostly coming from the Turonian beds of “Lastame” lithofacies of Scaglia Rossa Formation of northeastern Italy. Various specimens also come from upper Turonian to upper Campanian “Pietra di Castellavazzo” lithofacies (Scaglia Rossa Fm) indicating that these species might have lived for more than 10 million years, which is in contradiction with the assumption of Hamm (2020), who considers that the last occurrence of this species dates back to lower Coniacian. Younger occurrences of this taxon (middle Coniacian-Campanian) in Italy, however, remain dubious for the moment as long as the exact stratigraphic provenance of the “Castellavazzo” material is not firmly established. Considering the rarity of associated tooth sets, the two associated specimens (MCR FO 00662 and MCSNV v.1612) described herein provide additional significant information about this low-crowned species, which obviously was highly adapted to feed on hard-shelled prey. In particular, the well-preserved associated tooth set MCSNV v.1612 described in detail herein for the first time reveals crucial traits of dental variability within the lower dental plate, which are related to a marked heterodonty of *P. latissimus*. At least three different morphotypes (“s”, “l” and “d” in Fig. 13, see also above) are unambiguously recognizable by general tooth shapes and occlusal ornamentation patterns in MCSNV v.1612. In addition, the comparison with other low-crowned species known by articulated tooth sets allowed referring the teeth of associated tooth sets as well as the isolated teeth described herein to their original position within the dental plates. Consequently, intraspecific variability not related to the tooth position (e.g., symphyseal, lateral and distal) was also observed by re-examination of isolated teeth. Ontogeny-related and/or sex-related phenomena could explain some of these slight morphological variations (see also Hamm, 2010, 2017, 2020; Amadori et al., 2019b, 2020). Moreover, the isolated finds described herein reveal a degree of intraspecific variability similar to that observed in the associated specimens reported herein, supporting the validity of the observed morphotypes. However, it should be remarked that isolated teeth might represent, in some cases, elements from the upper dentition, which are not present in the associated tooth sets. Thus, further studies of more specimens are mandatory to properly define the overall dental configuration of *Ptychodus latissimus*.

The species *Ptychodus latissimus* was a third-level predator characterized by low-crowned, massive and sculptured teeth. Like other un-cuspidate species, *Ptychodus latissimus* was probably feeding predominantly on benthic, hard-shelled prey including the widespread inoceramids and ammonites using its very flat teeth with crested crown surfaces (see also Fossa-Mancini, 1921; Kauffman, 1972; Ozanne & Harries, 2002; Diedrich, 2013; Everhart, 2017; Amadori et al., 2020; Hamm, 2020).

## Supplemental Information

10.7717/peerj.10167/supp-1 Supplemental Information 1 Examined specimens of *Ptychodus*, housed in several museums in Northern Italy, in the Natural History Museum, London, in the Naturhistorisches Museum, Vienna. Some of these belong to different historical collections (C, ‘Catullo collection’; D, ‘Dixon collection’; M, ‘Mantell collection’; Z, ‘De Zigno Collection’).

10.7717/peerj.10167/supp-2 Supplemental Information 2 Measurements of associated teeth of *Ptychodus latissimus*, preserved in MCSNV v.1612; the find is housed in Museo Civico di Storia Naturale di Verona. CW, Crown width; CL, Crown length; CH, Crown height; nTR, number of transverse ridges; WT, worn tooth. Estimated values are indicated in parentheses.

10.7717/peerj.10167/supp-3 Supplemental Information 3 Measurements of teeth of isolated specimen of *Ptychodus latissimus* housed in several museums in Northern Italy and in the Naturhistorisches Museum, Vienna. CW = Crown width; CL = Crown length; CH = Crown height; RW = root width; RL = root length; RH = root height; nTR = number of transverse ridges; WT, worn tooth. Estimated values are indicated in parentheses.

10.7717/peerj.10167/supp-4 Supplemental Information 4 PCA parameters.

## Institutional abbreviations

MCV: Museo Civico ‘D. Dal Lago’, Vadagno (Vicenza), Italy
MCR: Museo Civico di Rovereto (Trento), Italy
MCSNV: Museo Civico di Storia Naturale di Verona, Italy
MSNUP: Museo di Storia Naturale dell’Università di Pisa, Italy
MGC VR: Museo Geopaleontologico di Camposilvano (Verona), Italy
MGP-PD: Museo di Geologia e Paleontologia dell’Università di Padova, Italy
NHMW: Naturhistorisches Museum, Vienna, Austria
NHMUK: Natural History Museum, London, UK

## Additional Information and Declarations

### Data Availability

The following information was supplied regarding data availability: Details on examined specimens, measurements of associated teeth preserved in MCSNV v.1612, measurements of isolated teeth and PCA parameters are available in the Supplemental Files.
